# Novel Anthra[1,2-c][1,2,5]Thiadiazole-6,11-Diones as Promising Anticancer Lead Compounds: Biological Evaluation, Characterization & Molecular Targets Determination

**DOI:** 10.1371/journal.pone.0154278

**Published:** 2016-04-21

**Authors:** Ahmed Atef Ahmed Ali, Yu-Ru Lee, Tsung-Chih Chen, Chun-Liang Chen, Chia-Chung Lee, Chia-Yang Shiau, Chiao-Hsi Chiang, Hsu-Shan Huang

**Affiliations:** 1 Molecular and Cell Biology, Taiwan International Graduate Program, Institute of Molecular Biology, Academia Sinica, Taipei, Taiwan; 2 Graduate Institute of Life Sciences, National Defense Medical Center, Taipei, Taiwan; 3 Graduate Institute for Cancer Biology and Drug Discovery, College of Medical Science and Technology, Taipei Medical University, Taipei, Taiwan; 4 Graduate Institute of Medical Sciences, National Defense Medical Center, Taipei, Taiwan; Islamic Azad University-Mashhad Branch, Mashhad, ISLAMIC REPUBLIC OF IRAN

## Abstract

The novel compounds NSC745885 and NSC757963 developed at our laboratory were tested against a panel of 60 cancer cell lines at the National Cancer Institute, USA, and a panel of 39 cancer cell lines at the Japanese Foundation of Cancer Research. Both compounds demonstrated selective unique multi-log differential patterns of activity, with GI_50_ values in the sub-micro molar range against cancer cells rather than normal cardiac cells. NSC757963 showed high selectivity towards the leukemia subpanel. Activities of both compounds strongly correlated to expression of NFKB1 and CSNK2B genes, implying that they may inhibit the NF-κB pathway. Immunocytochemical microscopy of OVCAR-3 cells showed clear cytosolic accumulation of the NF-κB p65 subunit following treatment. Western blotting showed dose dependent inhibition of the nuclear expression of the NF-κB p65 subunit with subsequent accumulation in the cytosol following treatment. Docking experiments showed binding of both compounds to the NF-κB activator IKKβ subunit preventing its translocation to the nucleus. Collectively, these results confirm the ability of our compounds to inhibit the constitutively active NF-κB pathway of OVCAR-3 cells. Furthermore, COMPARE analysis indicated that the activity of NSC757963 is similar to the antituberculosis agent rifamycin SV, this was confirmed by testing the antimycobacterial activity of NSC757963 against *Mycobacterium tuberculosis*, results revealed potent activity suitable for use in clinical practice. Molecular properties and Lipinski’s parameters predicted acceptable bioavailability properties with no indication of mutagenicity, tumorigenicity, irritability and reproductive effects. Oral absorption experiments using the human Caco-2 model showed high intestinal absorption of NSC745885 by passive transport mechanism with no intestinal efflux or active transport mechanisms. The unique molecular characterization as well as the illustrated anticancer spectra of activity and bioavailability properties warrant further development of our compounds and present a foundation brick in the pre-clinical investigations to implement such compounds in clinical practice.

## Introduction

**Fig 1:**
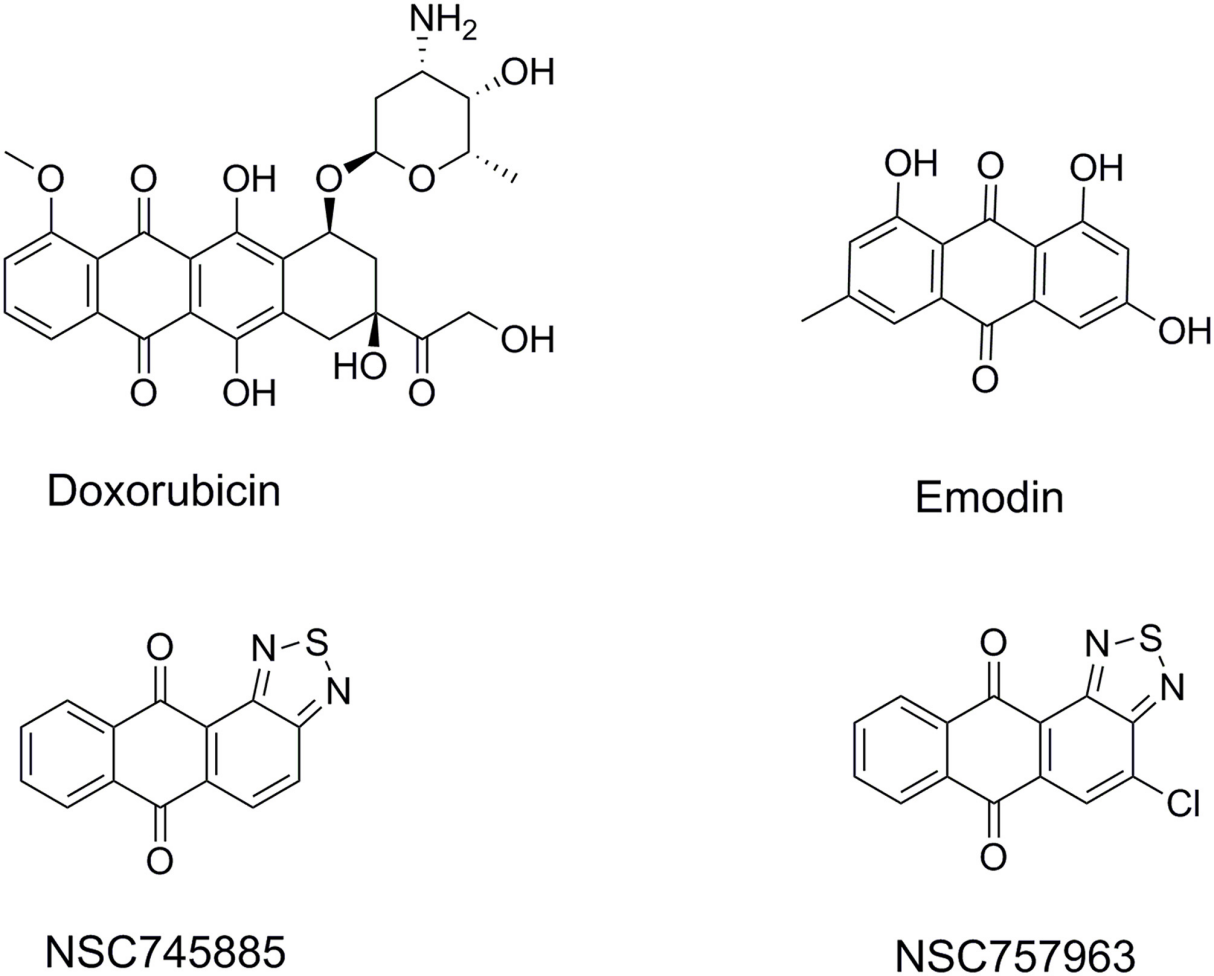
Representative anthraquinone and anthracycline structural sets used in the anticancer drug discovery and development.

The anthracycline antibiotics daunorubicin and doxorubicin, having the anthraquinone moiety, were introduced in clinical practice more than 30 years ago for treatment of a wide variety of cancers, but have limited use due to cardiotoxicity and bone marrow toxicity [1, 2]. Similarly, emodin is a natural product containing the anthraquinone moiety used in the folk medicine as anticancer and anti-inflammatory agent as well as inhibitor to the NF-κB pathway [3]. Therefore, the anthraquinone-based small molecules are regarded as promising lead candidates in cancer drug design if the adverse effects of their parent compounds are overcome with further development. As an attempt to develop more potent anthraquinone-based small molecules with less side effects than the parent drugs, we designed the 1,2-heteroannelated anthraquinone compounds namely NSC745885 (anthra[1,2-c][1,2,5]thiadiazole-6,11-dione) and its 4-chloro derivative NSC757963 presented in this study (Fig 1).

Primary screening of our library of compounds showed that NSC745885 exhibited higher telomerase inhibitory activity compared to the tested library members with potent cytotoxic effects [4]. Also, NSC745885 decreased expression of the tumor suppressor protein p53 and induced DNA damage in HeLa cells causing cells to arrest at sub-G_1_, these effects are different than what is observed with its parent drug doxorubicin [5]. In addition, NSC745885 overcame the resistance of the multi-drug resistance MGH-U1R cell line (40-fold resistance to doxorubicin) and showed completely suppressed growth of the resistant cell line by concentration as small as 2.5 μM [6]. This clearly implies that the mechanisms of action of NSC745885 is different from that of the parent drug doxorubicin, so we further investigated the novel mechanisms of action of NSC745885 in this study.

In this study, we aimed to describe the multidisciplinary evidences, approaches and experiments undertaken to systematically determine the cytotoxic spectra of activity and potential molecular targets of the novel lead molecules NSC745885 and NSC757963 using the full panel of the National Cancer Institute (NCI)-60 human tumor cell lines [non-small cell lung cancer, colon cancer, breast cancer, ovarian cancer, leukemia, renal cancer, melanoma, prostate cancer, and central nervous system (CNS) cancer], as well as the full panel of 39 human cancer cell lines of the Japanese Foundation for Cancer Research (JFCR-39) [breast, central nervous system (brain), melanoma, ovary, kidney, stomach and prostate], the relevant COMPARE analysis, and the *in vitro* experiments to confirm the obtained findings. Both compounds displayed highly unusual patterns of selectivity in the NCI-60 as well as in the JFCR-39 experiments with potent GI_50_ values in the sub-micro molar range. COMPARE analysis showed that both compounds may perform their cytotoxic activities through inhibiting the NF-κB pathway, a finding that was supported by the positive correlation between the activity of both compounds and the expression of NFKB1 and CSNK2B genes (encode the DNA binding subunit of the NF-κB protein complex and the beta subunit of casein kinase II (CK2) that activates the NF-κB pathway, respectively). Such findings were confirmed by the immunocytochemical imaging which showed that both compounds inhibited the translocation of the p65 subunit of the NF-κB from the cytosol to the nucleus as well as the Western blotting that showed inhibited expression of the NF-κB p65 subunit in the nuclear fractions of treated cells in a dose dependent manner with subsequent accumulation of the NF-κB p65 subunit in the cytosol, and docking studies which showed that both compounds may bind to IKKβ favorably, preventing the subsequent translocation of NF-κB to the nucleus. Collectively, these results confirm the ability of our compounds to inhibit the constitutively active NF-κB pathway. Furthermore, activity of both compounds was weakly correlated to the expression of the MGMT gene responsible for the resistance to chemotherapeutic drugs secondary to the activation of NF-κB. On the other side, COMPARE analysis showed that activity profile of NSC757963 is similar to that of the antituberculosis agent rifamycin SV, suggesting that our compound may exhibit antituberculosis activity. To confirm this interesting finding, we tested the antimycobacterial activity of NSC757963 against the *Mycobacterium tuberculosis* (H37Rv reference strain) and found the minimum inhibitory concentration (MIC) of NSC757963 to be 10 μg/mL, a concentration that is less than those of some antituberculosis drugs used in clinical practice [7], indicating the high potency and potential of our compound. We further supported both compounds to proceed to pre-clinical investigations by predicting their bioavailability and absorption—distribution—metabolism—elimination (ADME) properties, and found that both compounds may exhibit acceptable bioavailability and ADME properties with no indication of mutagenicity, tumorigenicity, irritability and reproductive effects. As a confirmation to the above findings, intestinal absorption experiments using the human Caco-2 cell permeability model [8, 9] showed that NSC745885 is highly absorbed through the intestinal cells, which was evident from the high absorptive permeability coefficient P_app(A→BL)_ = 31.8x10^-6^ cm/sec, the mechanism of absorption was found to be passive transport with no active transport or intestinal efflux mechanisms.

## Results and Discussion

### Cytotoxic activities of NSC745885 and NSC757963 obtained from single high dose & five dose testing on 60 human cancer cell lines (NCI) and 39 human cancer cell lines (JFCR)

**Fig 2:**
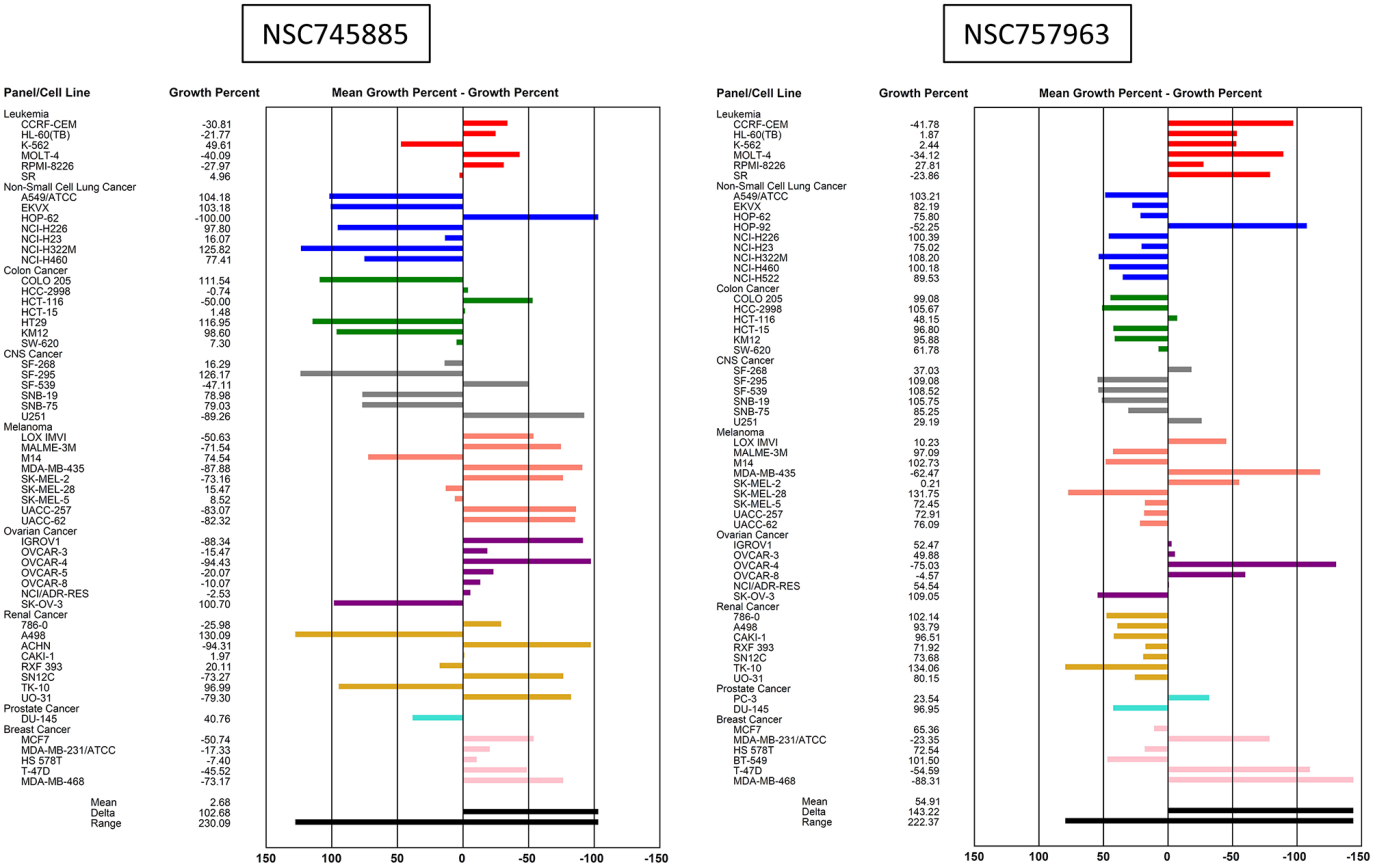
Sensitivity of the 60 human cancer cell lines to the cytotoxic activities of NSC745885 (left) and NSC757963 (right). Using a single dose of 10 μM of the test compound against the NCI 60 cell lines. Zero on the X-axis represents the mean percentage of growth of the tested cell lines. The percentage of growth of each cell line relative to the mean is represented by a horizontal bar extending to the right side indicating more sensitivity or to the left side indicating less sensitivity.

Results of the initial single dose (10 μM) testing of NSC745885 and NSC757963 against the 60 cell lines of NCI are presented in Fig 2. Activity of compounds is represented by the percentage of growth altered due to treatment. Melanoma cell lines were particularly sensitive to NSC745885. The highest activity for NSC745885 was 100% growth inhibition for the non-small cell lung cancer cell line HOP-62 followed by 94.43% growth inhibition for the ovarian cancer cell line OVCAR-4 and 94.31% growth inhibition for the renal cancer cell line ACHN. For NSC757963, the leukemia cell lines were the most affected. The highest growth inhibition was found to be 88.31% for the breast cancer MDA-MB-468 cell line.

Both NSC745885 and NSC757963 satisfied the pre-determined threshold inhibition criteria of the NCI-60 One-Dose Screening, so they were tested against the panels of 60 cancer cell lines of NCI (NCI-60) and 39 cancer cell lines of JFCR (JFCR-39), each cell line was tested at five small doses of our compounds. Results are expressed as the 50% growth inhibition concentration (GI_50_), the 50% lethal concentration (LC_50_) and the total growth inhibition (TGI), compared to the values of untreated control cells.

**Fig 3:**
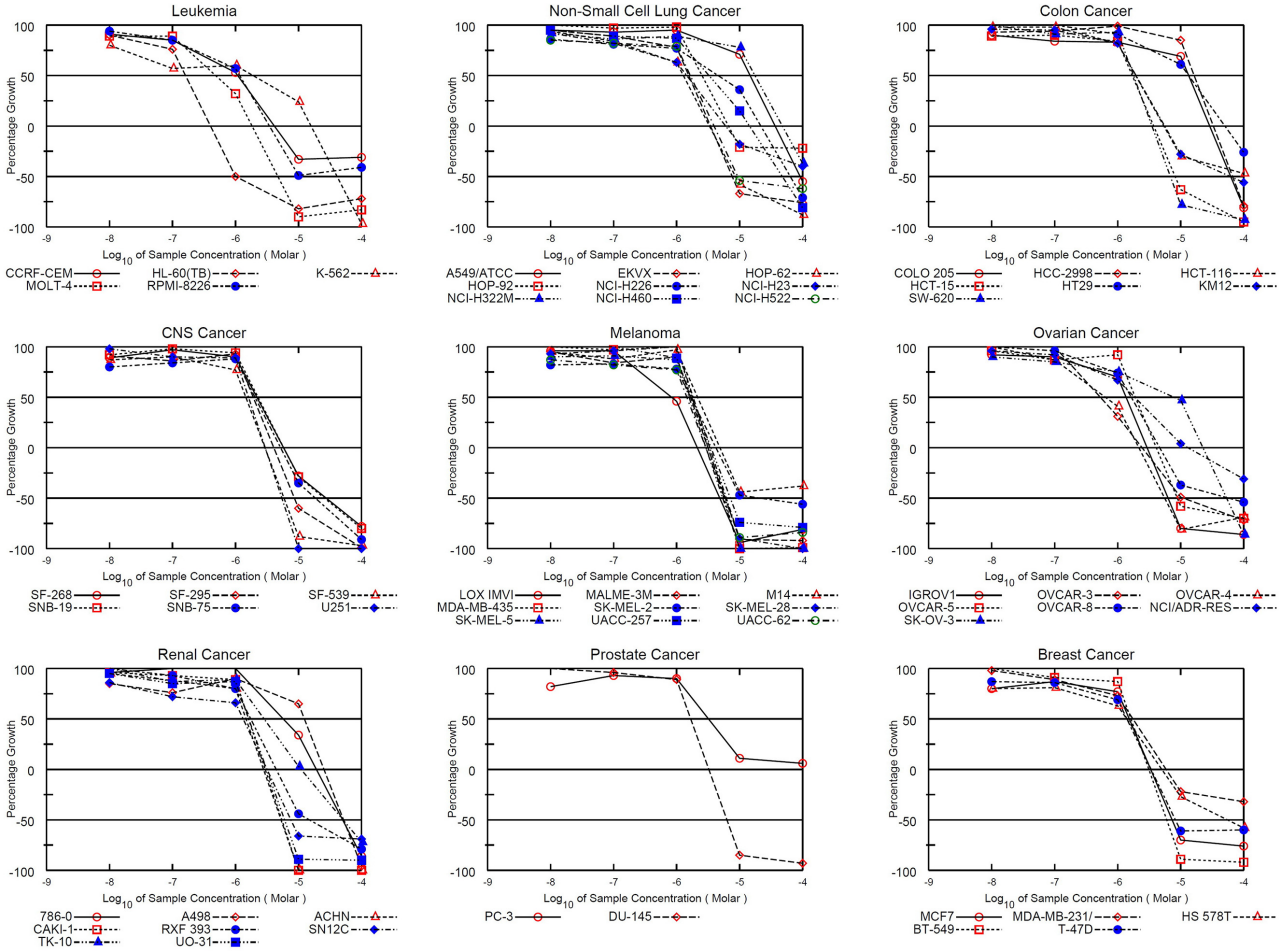
Dose response curves of the cytotoxic activity of NSC745885 against the NCI 60 human cancer cell lines. Growth percentage value of 100 represents the growth of untreated cells, while the value of 0 percentage represents no net growth throughout the period of the experiment, and the value of -100 indicates that all of the cells are killed at the end of the experiment.

**Fig 4:**
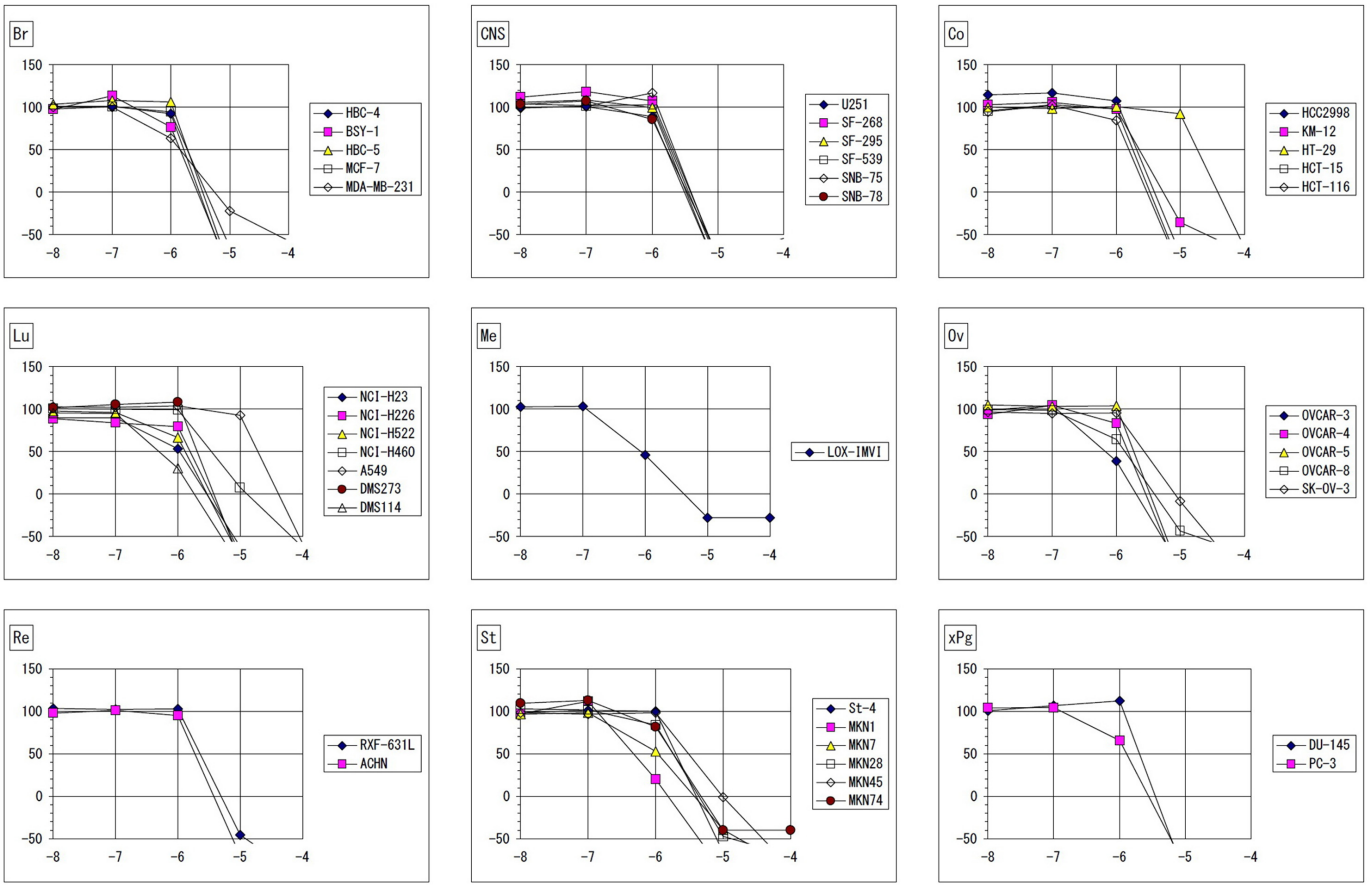
Dose response curves of the cytotoxic activity of NSC745885 against the JFCR 39 human cancer cell lines. X-axis represents log_10_ values (Molar) of the tested compound. Y-axis represents percentage of growth of the tested cells at the end of the experiment. Growth percentage value of 100 represents growth of the untreated cells, while the value of 0 percentage represents no net growth throughout the period of the experiment, and the value of -100 indicates that all cells are killed at the end of the experiment. Br: breast, CNS: central nervous system, Co: colon, Lu: lung, Me: melanoma, Ov: ovary, Re: renal, St: stomach, xPg: prostate.

Both NSC745885 and NSC757963 exhibited significant dose-dependent potent patterns of activity against most cancer cell lines. For NSC745885, the NCI-60 GI_50_ values ranged from 0.16 μM to 17.4 μM; the most sensitive cell line was the leukemia HL-60(TB) cell line showing a small sub-micro molar GI_50_ value of 0.16 μM followed by the leukemia MOLT-4, ovarian cancer OVCAR-3 and OVCAR-4, and melanoma LOX IMVI cell lines showing sub-micro molar GI_50_ values of 0.49, 0.55, 0.65 and 0.82 μM, respectively. The least growth inhibitory activity was for the non-Small cell lung cancer NCI-H322M cell line (GI_50_ = 17.4 μM) (Table 1, Fig 3 & S1 Fig). For the JFCR experiments, NSC745885 showed comparable results to those obtained from the NCI-60 experiments where it showed GI_50_ values ranging from 0.47 μM to 19 μM. The most sensitive cell line was the stomach cancer MKN1 cell line (GI_50_ = 0.47 μM) followed by the lung cancer DMS114, ovarian cancer OVCAR-3 and melanoma LOX-IMVI cell lines showing sub-micro molar GI_50_ values of 0.5, 0.67, and 0.86 μM, respectively. The least activity was for the colon cancer HT-29 cell line (GI_50_ = 19 μM) (Table 2, Fig 4 & S2 Fig).

**Table 1:** Cytotoxic activities of NSC745885 and NSC757963 against the NCI 60 human cancer cell lines.

Panel/Cell line	NSC745885 (μM)			NSC757963 (μM)		
	GI_50_	TGI	LC_50_	GI_50_	TGI	LC_50_
**Leukemia**						
CCRF-CEM	1.08	4.10	>100	0.33	2.37	>100
HL-60(TB)	0.16	0.39	0.99	0.42	2.22	>100
K-562	1.92	15.9	41.2	-	-	-
MOLT-4	0.49	1.84	4.71	0.46	26.7	>100
RPMI-8226	1.18	3.46	>100	3.15	53.2	>100
SR	-	-	-	2.64	>100	>100
**Non-small cell lung cancer**						
A549/ATCC	14.7	36.6	91.0	>100	>100	>100
EKVX	1.55	3.45	7.66	11.0	23.2	48.7
HOP-62	1.28	3.31	8.53	4.95	17.3	43.3
HOP-92	2.52	6.64	>100	1.43	3.34	7.84
NCI-H226	4.48	21.6	63.7	14.9	28.3	53.9
NCI-H23	1.44	6.04	>100	3.62	17.2	74.6
NCI-H322M	17.4	47.8	>100	18.6	36.3	71.0
NCI-460	3.27	14.3	47.3	30.2	>100	>100
NCI-H522	1.66	3.93	9.31	1.6	3.62	8.18
**Colon cancer**						
COLO 205	13.4	28.9	62.4	16.9	31.2	57.9
HCC-2998	16.4	33.1	66.6	14.1	28.2	56.6
HCT-116	1.93	5.40	>100	3.45	11.8	35.3
HCT-15	1.71	3.72	8.12	10.2	23.8	55.9
HT29	13.4	50.4	>100	19.2	43.4	98.2
KM12	1.96	5.54	59.7	12.9	25.9	52.1
SW-620	1.79	3.50	6.84	2.87	10.4	58.7
**CNS cancer**						
SF-268	2.17	5.77	27.7	2.07	4.83	13.9
SF-295	1.90	4.04	8.56	16.6	32.3	62.9
SF-539	1.46	2.94	5.91	8.23	21.6	50.8
SNB-19	2.28	5.82	26.0	13.9	28.6	58.9
SNB-75	2.03	5.20	18.6	2.17	6.89	69.1
U251	1.61	2.96	5.44	3.29	10.4	32.7
**Melanoma**						
LOX IMVI	0.82	2.13	4.86	1.91	5.21	19.7
MALME-3M	1.66	3.15	5.96	15.9	30.7	59.1
M14	2.15	4.85	>100	17.0	31.9	59.7
MDA-MB-435	1.91	3.31	5.76	1.99	3.86	7.49
SK-MEL-2	1.68	4.22	22.5	2.21	5.77	37.7
SK-MEL-28	1.85	3.38	6.18	9.52	21.5	47.0
SK-MEL-5	1.58	2.92	5.41	8.48	21.0	46.2
UACC-257	1.74	3.51	7.09	14.5	27.8	53.5
UACC-62	1.45	2.90	5.79	4.43	17.0	41.8
**Ovarian cancer**						
IGROV1	1.36	2.94	6.35	3.3	15.6	45.7
OVCAR-3	0.55	2.46	11.1	1.74	3.23	5.99
OVCAR-4	0.65	2.17	5.57	1.46	2.83	5.5
OVCAR-5	1.91	4.11	8.84	17.6	31.6	56.8
OVCAR-8	1.66	4.68	59.8	2.39	5.19	>100
NCI/ADR-RES	1.86	12.6	>100	3.12	18.8	>100
SK-OV-3	7.71	22.5	53.4	17.8	32.1	58.0
**Renal cancer**						
786–0	6.17	19.3	50.4	17.2	31.8	58.8
A498	12.4	25.4	52.0	72.8	>100	>100
ACHN	1.46	2.77	5.27	2.0	3.93	7.74
CAKI-1	1.61	2.96	5.44	12.3	26.8	58.8
RXF 393	1.75	4.40	14.6	2.17	7.21	27.1
SN12C	1.33	3.17	7.56	3.27	13.1	36.1
TK-10	2.85	10.9	50.4	24.1	39.3	64.1
UO-31	1.62	3.12	6.01	14.0	27.1	52.4
**Prostate Cancer**						
PC-3	3.23	>100	>100	2.85	30.2	>100
DU-145	1.67	3.24	6.27	14.2	27.2	52.2
**Breast cancer**						
MCF7	1.53	3.34	7.29	1.78	4.24	10.4
MDA-MB-231/ATCC	1.75	5.90	>100	1.32	3.37	8.63
HS 578T	1.41	5.02	56.0	2.38	-	>100
BT-549	1.63	3.13	6.01	18.1	32.7	58.9
T-47D	1.39	3.39	8.27	1.97	4.56	>100
MDA-MB-468	-	-	-	1.38	2.93	6.25

**Table 2:** Cytotoxic activities of NSC745885 and NSC757963 against the JFCR 39 human cancer cell lines.

Panel/Cell line	NSC745885 (μM)			NSC757963 (μM)		
	GI_50_	TGI	LC_50_	GI_50_	TGI	LC_50_
**Breast Cancer**						
HBC-4	1.7	3.0	5.5	1.7	3.1	5.6
BSY-1	1.4	2.8	5.6	1.7	3.2	6.0
HBC-5	1.9	3.5	6.2	2.1	4.1	8.3
MCF-7	1.9	3.9	8.1	1.8	3.9	8.0
MDA-MB-231	1.4	5.5	63.0	1.3	2.9	6.4
**CNS cancer**						
U251	1.6	3.1	5.8	1.9	3.9	8.1
SF-268	2.0	3.8	7.1	2.0	4.0	6.5
SF-295	1.8	3.3	5.9	1.9	3.5	6.5
SF-539	1.9	3.6	6.6	1.9	3.4	6.4
SNB-75	2.2	3.9	7.0	2.1	3.8	6.8
SNB-78	1.7	3.4	6.9	2.2	4.1	7.9
**Colon cancer**						
HCC2998	2.1	3.9	7.5	1.9	3.5	6.4
KM-12	2.3	5.4	25.0	3.0	8.4	36.0
HT-29	19.0	39.0	81.0	21.0	45.0	98.0
HCT-15	1.8	3.4	6.3	1.9	3.9	7.9
HCT-116	1.6	3.0	5.8	1.9	4.0	8.3
**Lung cancer**						
NCI-H23	1.1	2.9	8.0	1.9	4.1	8.7
NCI-H226	1.6	3.4	7.3	4.2	17.0	51.0
NCI-H522	1.3	3.0	6.7	1.8	3.6	7.3
NCI-H460	3.4	13.0	67.0	10.0	26.0	64.0
A549	19.0	41.0	89.0	20.0	43.0	95.0
DMS273	2.0	3.8	7.0	2.1	4.1	8.1
DMS114	0.5	1.8	5.0	1.0	2.5	6.1
**Melanoma**						
LOX-IMVI	0.86	4.2	>100	1.9	4.2	9.3
**Ovarian cancer**						
OVCAR-3	0.67	2.0	5.1	1.9	4.2	9.1
OVCAR-4	1.5	2.9	5.5	1.6	3.0	5.6
OVCAR-5	1.9	3.4	6.0	1.6	3.0	5.5
OVCAR-8	1.4	4.0	18.0	2.1	4.8	>100
SK-OV-3	2.7	8.3	29.0	3.2	14.0	38.0
**Renal cancer**						
RXF-631L	2.3	4.9	12.0	1.7	3.1	5.9
ACHN	1.9	3.8	7.6	1.8	3.4	6.5
**Stomach cancer**						
ST-4	2.0	4.0	8.0	2.0	3.9	7.4
MKN1	0.47	1.5	4.3	1.8	3.4	6.6
MKN7	1.1	3.7	17.0	1.7	3.6	7.4
MKN28	1.8	4.4	13.0	2.2	4.4	8.6
MKN45	3.1	9.8	39.0	2.0	5.1	17.0
MKN74	1.8	4.7	>100	2.1	4.2	8.4
**Prostate Cancer**						
DU-145	2.0	3.5	6.1	2.5	6.3	23.0
PC-3	1.3	2.8	6.0	1.6	3.2	6.5

**Fig 5:**
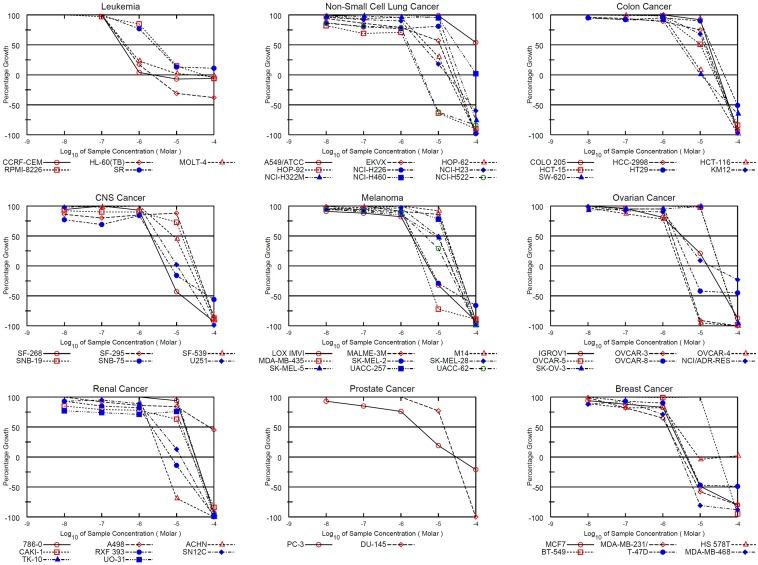
Dose response curves of the cytotoxic activity of NSC757963 against the NCI 60 human cancer cell lines. Growth percentage value of 100 represents the growth of untreated cells, while the value of 0 percentage represents no net growth throughout the period of the experiment, and the value of -100 indicates that all of the cells are killed at the end of the experiment.

**Fig 6:**
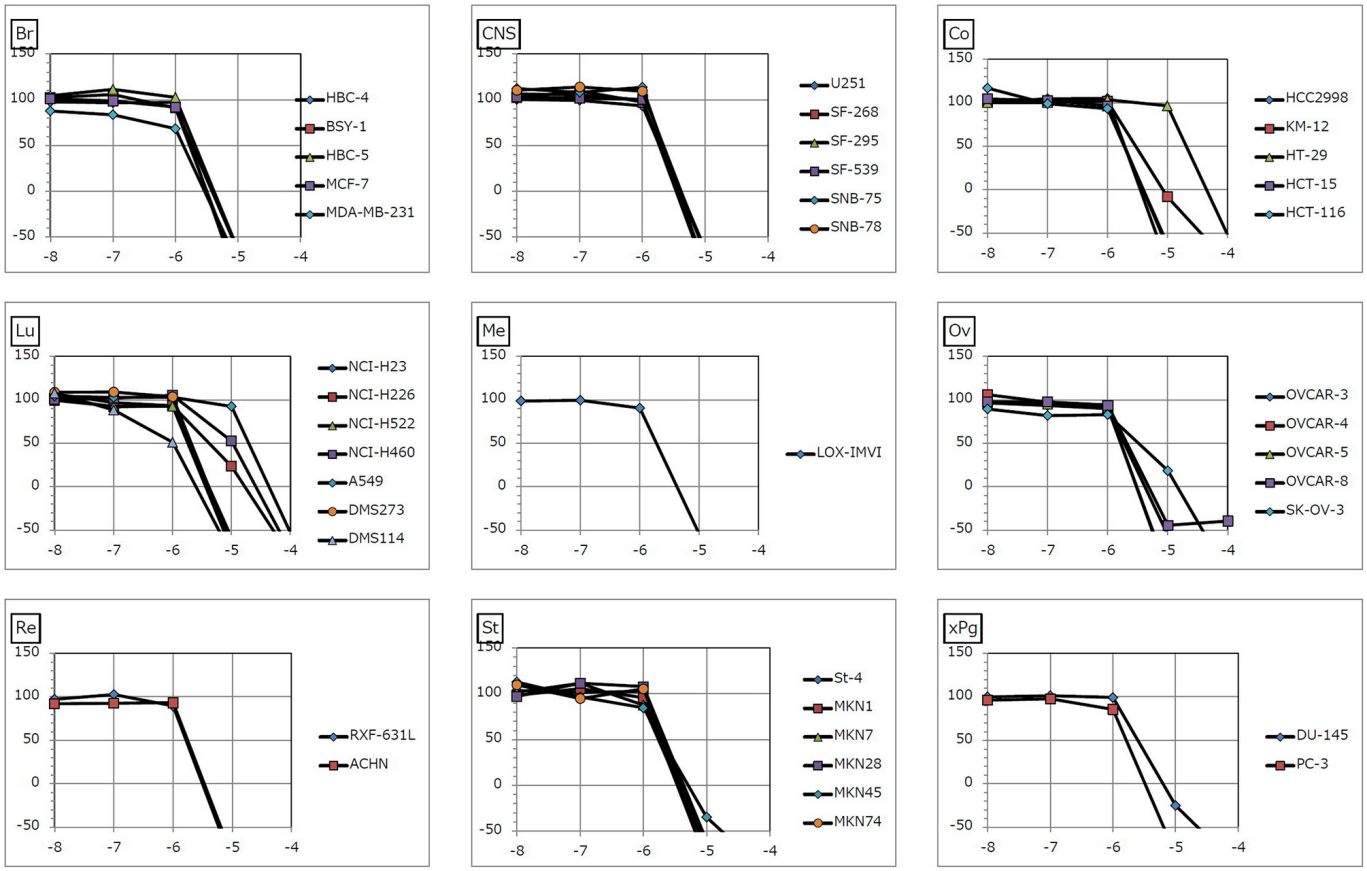
Dose response curves of the cytotoxic activity of NSC757963 against the JFCR 39 human cancer cell lines. X-axis represents log_10_ values (Molar) of the tested compound. Y-axis represents percentage of growth of the tested cells at the end of the experiment. Growth percentage value of 100 represents growth of the untreated cells, while the value of 0 percentage represents no net growth throughout the period of the experiment, and the value of -100 indicates that all cells are killed at the end of the experiment. Br: breast, CNS: central nervous system, Co: colon, Lu: lung, Me: melanoma, Ov: ovary, Re: renal, St: stomach, xPg: prostate.

For NSC757963, the NCI-60 GI_50_ values ranged from 0.33 μM to 72.8 μM with the exception of one non-small cell lung cancer cell line (A549/ATCC), which showed a GI_50_ value of more than 100 μM. The leukemia cell lines showed a significant sensitivity to the compound; three out of five of the tested cell lines namely CCRF-CEM, HL-60(TB) and MOLT-4 showed sub-micro molar GI_50_ values as small as 0.33, 0.42 and 0.46 μM, respectively (Table 1, Fig 5 & S3 Fig). For the JFCR-39 screening, NSC757963 showed GI_50_ values ranging from 1 μM to 21 μM. The most sensitive cell line was the lung cancer DMS114 cell line (GI_50_ = 1 μM). The least activity was for the colon cancer HT-29 cell line (GI_50_ = 21 μM) and the lung cancer A549 cell line (GI_50_ = 20 μM) (Table 2, Fig 6 & S4 Fig). We observed from the five-dose NCI-60 and JFCR-39 results that both lead compounds exhibit multi-log differential potent patterns of activity, where NSC745885 is specifically effective against ovarian cancer and melanoma. On the other side, lung cancer may have resistant cell lines towards NSC745885 and NSC757963.

### Cytotoxicity to normal cardiac cells and selectivity among cancer types of NSC745885 and NSC757963

Despite the potent anticancer activity of doxorubicin, its cardiotoxicity limits its use in clinical practice [1, 2]. We designed NSC745885 and NSC757963 containing the anthraquinone core structure similar to doxorubicin, so we aimed to determine the cytotoxicity of our new compounds towards normal cardiac myoblast H9c2 cells compared to doxorubicin (S5 Fig). Doxorubicin showed potent dose dependent cytotoxic effect on H9c2 cells. However, both NSC745885 and NSC757963 showed significantly less cytotoxic effects on H9c2 cells at higher doses than doxorubicin, indicating that our compounds may achieve the therapeutic concentrations without being cytotoxic to cardiac cells. Also, these results indicate that NSC745885 and NSC757963 exhibit specificity towards cancer cells rather than normal cells.

To characterize the selectivity of NSC745885 and NSC757963 further among the different types of cancer, we determined their selectivity ratios towards each cell line subpanel used in the cytotoxicity experiments [10]. NSC745885 showed a broad-spectrum inhibitory activity on the tested NCI cancer cell line subpanels which is evident from the selectivity ratios of values less than 3 for all NCI 60 cell line subpanels (except for the leukemia cell line subpanel where it showed moderate selectivity with a selectivity ratio of 3.35). Along the same line, this compound showed broad-spectrum inhibitory activity on the tested JFCR cell line subpanels; the selectivity ratios of all JFCR subpanels were below 3 except for the melanoma subpanel where it showed moderate selectivity with a selectivity ratio of 3.04 (S1 and S2 Tables). The promising activity of this compound is evident from the small mean GI_50_ values. The mean GI_50_ values of all the NCI 60 cell line subpanels ranged from 0.97 to 7.2 μM. The strongest activity was towards the leukemia subpanel and the least one was towards the colon cancer subpanel. Similarly, the mean GI_50_ values of all the JFCR cell line subpanels ranged from 0.86 to 5.36 μM. The strongest activity was towards the melanoma subpanel and the least one was towards the colon cancer subpanel.

NSC757963 showed high selectivity towards the NCI leukemia subpanel, which is evident from the high selectivity ratio of (7.79). It showed also a broad-spectrum inhibitory activity against the other NCI subpanels; the selectivity ratios of other NCI subpanels were less than 3. Similar broad-spectrum activity was observed among the JFCR subpanels where all of the tested subpanels showed selectivity ratio values of less than 3. Furthermore, NSC757963 showed remarkable growth inhibitory activity, which is evident from the low mean GI_50_ values of the NCI subpanels ranging from 1.4 to 20.7 μM and of the JFCR subpanels ranging from 1.72 to 5.9 μM (S1 and S2 Tables).

### Determining the molecular targets & classes of therapeutics of NSC745885 and NSC757963

Compounds having similar activity profiles (fingerprints) usually have similar mechanisms of action and modes of resistance. A method named “COMPARE analysis” was developed to measure the degree of correlation between the compounds of interest and the known drugs as well as the known molecular targets in the NCI databases using the Pearson’s correlation coefficient (PCC) determination as its principle [11]. The PCC values range from -1.0 (perfect negative correlation) to 1.0 (perfect positive correlation), where values in between determines the degree of correlation, and a value of zero means there is no correlation [12].

In order to identify the molecular targets that may account for the activity of our compounds, we mined the NCI “Molecular Targets” database (gene mutations, mRNA levels, protein levels and enzyme activities) to correlate the expression of such targets to the activity of our compounds. We identified multiple molecular targets that correlated strongly with our compounds (positive and negative correlations were identified). Results showed that the activity of NSC745885 is positively correlated to the expression of the following genes: ZNF184, NFKB1, PDHB, ARMC10, ZXDC, SYNCRIP, NXF1, RING1, CSNK2B and CDC5L with PCC values ≤ 0.566, while its activity is correlated to the suppression of the following genes: S100A10, PPP2R2C, PCSK6, TXN, TRIM16, EIF4E, CIB1, NXN, GCLC and CDC14B with PCC values ≤ -0.672 (more information about these genes and their degrees of correlation to the activity of NSC745885 are found in Table 3).

**Table 3:** Molecular targets correlated to NSC745885 activity.

Pearson’s correlation coefficient ^a^	Genecard code	Molecular target ID	Description
0.566	ZNF184	GC97384	zinc finger protein 184
0.551	NFKB1	GC11933	nuclear factor of kappa light polypeptide gene enhancer in B-cells 1
0.497	PDHB	GC164686	pyruvate dehydrogenase (lipoamide) beta
0.493	ARMC10	GC51034	armadillo repeat containing 10
0.481	ZXDC	GC48490	ZXD family zinc finger C
0.475	SYNCRIP	GC220136	synaptotagmin binding, cytoplasmic RNA interacting protein
0.449	NXF1	GC13658	nuclear RNA export factor 1
0.449	RING1	GC13087	ring finger protein 1
0.425	CSNK2B	GC236426	casein kinase 2, beta polypeptide
0.407	CDC5L	GC228762	CDC5 cell division cycle 5-like (*S*. *pombe*)
-0.672	S100A10	GC199093	S100 calcium binding protein A10
-0.63	PPP2R2C	GC209854	protein phosphatase 2, regulatory subunit B, gamma
-0.604	PCSK6	GC195349	proprotein convertase subtilisin/kexin type 6
-0.579	TXN	GC152424	thioredoxin
-0.578	TRIM16	GC56470	tripartite motif containing 16
-0.573	EIF4E	GC9932	eukaryotic translation initiation factor 4E
-0.566	CIB1	GC184632	calcium and integrin binding 1 (calmyrin)
-0.553	NXN	GC17230	nucleoredoxin
-0.552	GCLC	GC176620	glutamate-cysteine ligase, catalytic subunit
-0.551	CDC14B	GC176787	CDC14 cell division cycle 14 homolog B (*S*. *cerevisiae*)

^a^ The range of this coefficient is from -1 to +1. Positive values approaching 1 indicate high positive correlation between the test compound and the gene expression, while those with negative coefficient values approaching -1 indicate high negative correlation between the test compound and the gene expression, and a value of zero indicates no correlation at all.

Results showed that the activity of NSC745885 has strong positive correlation to the expression of NFKB1 gene with a PCC value of 0.551; this gene encodes a protein that is a DNA binding subunit of the NF-κB protein complex. Activation of NF-κB enhances malignant transformation and tumor progression as well as assisting the tumor cells to escape the immune surveillance, this clarified the importance of inhibiting the activation of NF-κB as an anticancer treatment strategy [13]. The identified correlation strongly suggest that NSC745885 is an NF-κB inhibitor either by direct or indirect influences which is in concordance with the previously published data concerning the efficiency of the anthraquinone moiety in inactivating the NF-κB pathway [3]. Furthermore, NSC745885 was positively correlated to the expression of CSNK2B gene (encodes the beta subunit of the CK2), which plays an important role in activating the NF-κB. It was found that knockdown of CK2 subunits inhibited IκB degradation, nuclear translocation of NF-κB and its DNA binding in cancer cells [14]. Collectively, these findings suggest that our compounds may inhibit the activity of NF-κB pathway by inhibiting its translocation to nucleus by direct or indirect ways. We further confirmed these findings as explained later in this study.

Along the same line, activity of NSC757963 was positively correlated to the expression of the following genes: STMN1, ZNRD1, JARID2, ARHGAP19, MEN1, RPL5, TFDP2, NFKB1, PRPF4B and ERBB2 with PCC values ≤ 0.632, while its activity was correlated to the suppression of the following genes: EPHX1, NQO1, ABCC3, THAP10, PTGR1, MGAT4B, AKR1C3, ALDH3A1, NXN and SRC with PCC values ≤ -0.7 (more information about these genes and their degrees of correlation to the activity of NSC757963 are found in Table 4).

**Table 4:** Molecular targets correlated to NSC757963 activity.

Pearson’s correlation coefficient ^a^	Genecard code	Molecular target ID	Description
0.632	STMN1	MT18314	stathmin 1
0.579	ZNRD1	GC214345	zinc ribbon domain containing 1
0.568	JARID2	GC234506	jumonji, AT rich interactive domain 2
0.564	ARHGAP19	GC27981	Rho GTPase activating protein 19
0.555	MEN1	GC15296	multiple endocrine neoplasia I
0.552	RPL5	GC36655	ribosomal protein L5
0.541	TFDP2	GC89284	transcription factor Dp-2 (E2F dimerization partner 2)
0.534	NFKB1	GC11933	nuclear factor of kappa light polypeptide gene enhancer in B-cells 1
0.519	PRPF4B	GC148097	PRP4 pre-mRNA processing factor 4 homolog B (yeast)
0.508	ERBB2	MT10651	v-erb-b2 erythroblastic leukemia viral oncogene homolog 2, neuro/glioblastoma derived oncogene homolog (avian)
-0.7	EPHX1	GC235776	epoxide hydrolase 1, microsomal (xenobiotic)
-0.639	NQO1	GC227340	NAD(P)H dehydrogenase, quinone 1
-0.623	ABCC3	GC28671	ATP-binding cassette, sub-family C (CFTR/MRP), member 3
-0.606	THAP10	GC12979	THAP domain containing 10
-0.606	PTGR1	GC209176	prostaglandin reductase 1
-0.603	MGAT4B	GC72511	mannosyl (alpha-1,3-)-glycoprotein beta-1,4-N-acetylglucosaminyltransferase, isozyme B
-0.597	AKR1C3	GC27801	aldo-keto reductase family 1, member C3 (3-alpha hydroxysteroid dehydrogenase, type II)
-0.593	ALDH3A1	MT198	aldehyde dehydrogenase 3 family, member A1
-0.564	NXN	GC17230	nucleoredoxin
-0.514	SRC	MT819	v-src sarcoma (Schmidt-Ruppin A-2) viral oncogene homolog (avian)

^a^ This coefficient ranges from -1 to +1. Positive values approaching 1 indicate high positive correlation between the test compound and gene expression, while those with negative coefficient values approaching -1 indicate high negative correlation between the test compound and gene expression, and a value of zero indicates no correlation at all.

**Fig 7:**
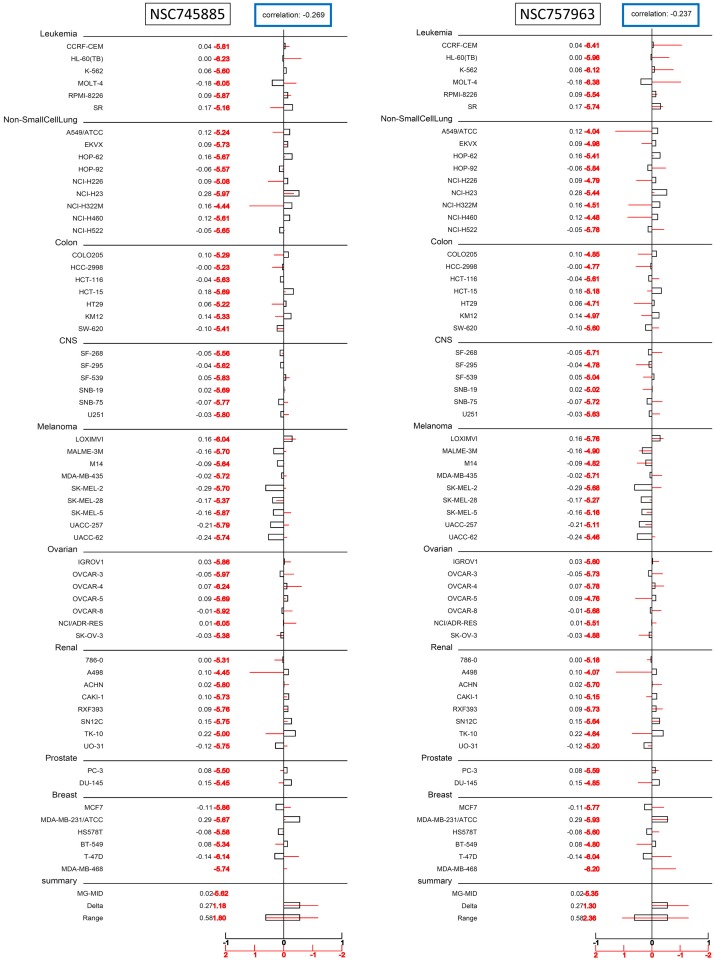
Correlation of the activity of NSC745885 (left) and NSC757963 (right) to the expression of MGMT gene obtained from the NCI 60 cell line experiments. Values and bars in red color represent the activities of the compounds towards each tested cell line (mean log_10_ of the GI_50_ values), while those in black color represent the expression level of the gene in each cell line. Total Pearson’s Correlation Coefficient values are displayed on top of the figure, where it ranges from -1 to +1; positive values approaching 1 indicate high positive correlation between the test compound and the gene expression, while those with negative values approaching -1 indicate high negative correlation, and a value of zero indicates no correlation at all.

Activity of NSC757963, similar to that of NSC745885, has a strong positive correlation to the expression of NFKB1 gene (its role has been mentioned earlier) with a PCC value of 0.534. Expression of NFKB1 gene is associated with the resistance to several anticancer agents with different mechanisms of action [15]. The mechanism of this chemoresistance is mediated through the over expression of MGMT gene (*O*^*6*^-methylguanine DNA methyl transferase). MGMT is a cellular DNA repair protein that restores the damaged DNA to its normal state [16]. It has been proved that MGMT is a target gene for the NF-κB and hence activation of NFKB1 gene will increase the expression of MGMT leading to resistance to the anticancer agent [17]. These findings raised an important issue, whether both compounds (that correlates positively to the expression of NFKB1) will suffer from resistance by this mechanism? To answer this question, we tested the correlation between the activity fingerprints of both compounds and the expression of MGMT gene. Results showed that expression of the MGMT gene is weakly correlated to the activity of both compounds (Fig 7). The PCC values of correlation between the expression of MGMT gene and the activities of NSC745885 and NSC757963 were -0.269 and -0.237, respectively, indicating that the activities of both compounds were nearly not affected by the expression of MGMT gene. These findings are in concordance with our previous finding that NSC745885 could achieve potent growth inhibition to the multi-drug resistant MGH-U1R cell line using the same concentration that inhibited the non-resistant MGH-U1 cells (2.5 μM) [6]. This assures the promising potential of both compounds as anticancer agents against the multi-drug resistant tumors.

Regarding the NCI Standard Agents, results showed that NSC745885 has positive correlations mostly with alkylating agents (S3 Table); the top five compounds correlated with their GI_50_ fingerprint were alkylating agents, with PCC values ranging from 0.451 to 0.382. The highest two correlated compounds were melphalan and asaley. The correlations of both melphalan and asaley were further supported with the results of the TGI fingerprint COMPARE analysis where both compounds ranked in the top ten correlations, indicating that the inhibitory and cytostatic activities of NSC745885 may be similar to those of melphalan and asaley. Moreover, the cytostatic activity of NSC745885 strongly correlated to the alkylating agent dianhydrogalactitol showing a TGI-PCC value of 0.547. On the other side, the first negative correlation with the TGI fingerprint was with N,N-dibenzyl daunomycin which is a topoisomerase II Inhibitor. This observation supports our molecular docking results which revealed that NSC745885 has low affinity for topoisomerase IIA compared to doxorubicin [5].

Along the same line, NCI-COMPARE analysis of NSC745885 using the Marketed Drugs database revealed that it has positive correlation to melphalan with a PCC value of 0.451. The second positive correlation was with trisenox (arsenic trioxide) with a PCC value of 0.423. For the negative correlations, calcium leucovorin and actinomycin D showed the highest negative correlations with PCC values of -0.419 and -0.411, respectively (S4 Table). On the other side, results of the JFCR-COMPARE analysis showed that NSC745885 did not correlate to alkylating agents where it exhibited weak positive correlations with bleomycin, indisulam and cisplatin with PCC values of 0.417, 0.408 and 0.39, respectively (S5 Table).

NCI-COMPARE analysis of NSC757963 using the Standard Agents database (S6 Table) revealed that compounds with mechanisms of action different from those correlated to NSC745885 emerged to correlate strongly to NSC757963; 5-hydroxypicolinaldehyde thiosemicarbazone (5HP) and diglycoaldehyde, the DNA antimetabolites showed GI_50_ PCC values of 0.552 and 0.48, respectively. Another DNA antimetabolite agent, macbecin II, showed strong positive correlation to our compound with an LC_50_ PCC value of 0.51. Interestingly, rifamycin SV showed high positive correlation to NSC757963 with a GI_50_ PCC value of 0.535. Moreover, the cytotoxic and cytostatic profiles of rifamycin SV correlated positively to those of NSC757963 with PCC values of 0.561 and 0.458 for the LC_50_ and TGI fingerprints. This strong correlation in the activity profiles imply that NSC757963 may have similar mechanism of action to rifamycin SV and hence may have similar uses to it. Rifamycin SV is a natural antibiotic that has proven superior potency against *Mycobacterium spp*., long term treatment with this drug, which is typically the case for tuberculosis patients, may induce hepatotoxicity and so, new drugs with comparable activities and less adverse effects are necessary to be developed [18]. This need together with the correlation data of NSC757963 and the emergence of both multidrug-resistant and extensively drug-resistant tuberculosis [19, 20], may open the door for new research direction for NSC757963 as a safer and potent anti-tuberculosis agent. As a first step to develop NSC757963 as anti-tuberculosis agent and confirm its activity, we tested its antimycobacterial activity as discussed later in this article. Other important correlations of NSC757963 were between the cytotoxic and cytostatic activities of NSC757963 and alkylating agents; analysis of LC_50_ showed PCC values of 0.557 and 0.549 with asaley and diaziquone (AZQ) and showed GI_50_ PCC values of 0.523, 0.495 and 0.491 with fluorodopan, semustine (methyl CCNU) and asaley, respectively.

We noted that JFCR-COMPARE analysis results of NSC757963 also ranked an alkylating agent as the first positive correlation; however, the PCC value (0.307) was less than those obtained from the NCI analysis, and the highest correlated alkylating agent from the JFCR database (4-hydroperoxycyclophosphamide) was different from the correlated agents of the NCI databases. Other correlated agents were indisulam and 6-mercaptopurine with PCC values of 0.301 and 0.260, respectively. (S7 Table)

The differences observed between the NCI and JFCR COMPARE analysis results may be due to the difference in their databases. The NCI database includes a greater number of compounds and more database sets than the JFCR, to use in the COMPARE analysis (this may account for the more number of correlated alkylating agents which we identified from mining the NCI database than from the JFCR database). Moreover, the NCI uses the “60 cell line fingerprint” which applies more data to run the COMPARE analysis than that of the “JFCR 39 cell line fingerprint” to get more accurate and fine-tuned results. Although the JFCR 39 database might cover most of the possible molecular targets [21], and proved to be effective in identifying new anticancer agents [22], any reduction in the number of compared cell lines will reduce the accuracy and decrease the resolution of the comparison [23].

### NSC745885 and NSC757963 suppress constitutive NF-κB activation by inhibiting its translocation to the nucleus

The transcription factor NF-κB plays a key role in inflammation, apoptosis and oncogenesis. Many cancer cells exhibit constitutive NF-κB activation, and inhibiting such activity results in the cell death, a mechanism that can be used to treat cancer [24]. NF-κB is a heterodimeric complex of Rel proteins including the RelA (p65) subunit that is one of the most prevalent dimer subunits found in cells [24]. NF-κB is typically residing in the cytosol in its inactive state bound to the inhibitory proteins of IκB family. Upon activation of the NF-κB pathway, IκB is phosphorylated and degraded allowing the NF-κB to translocate to the nucleus and bind to promoter-specific κB consensus DNA elements initiating the transcription of diverse array of NF-κB target genes [25–27].

**Fig 8:**
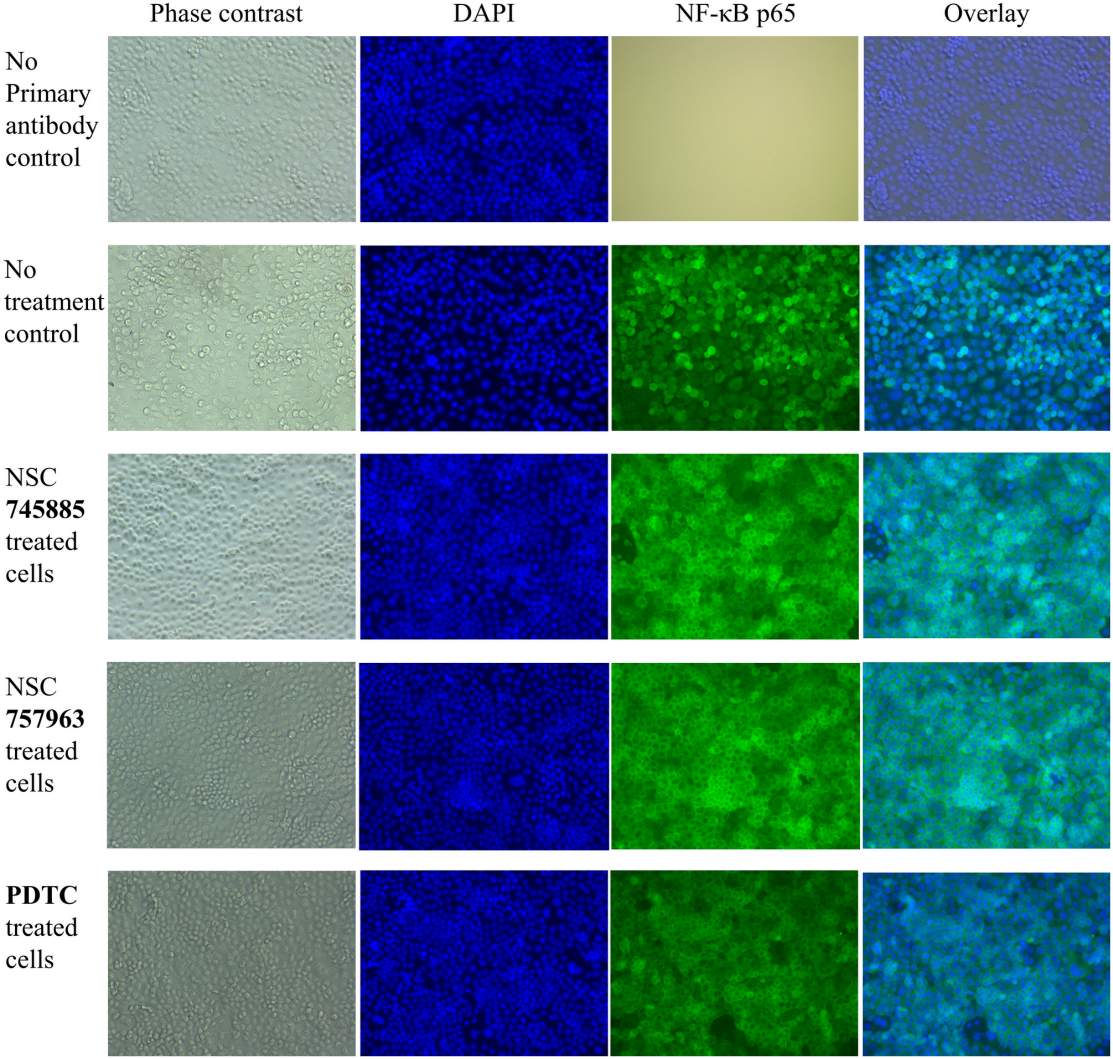
Inhibition of the NF-κB activity of OVCAR-3 cells treated with NSC745885 or NSC757963. Cells were treated with either NSC745885 or NSC757963 prior to the immunocytochemical staining with NF-κB p65 primary antibody followed by Alexa Fluor^®^ secondary antibody. Nuclei of the cells were stained with DAPI. Accumulation of the p65 subunit (green) at the cytosol rather than the nucleus (blue) indicates inhibition to the constitutively active NF-κB pathway of OVCAR-3 cells. Three control experiments are presented; positive treatment control using PDTC as a known chemical inhibitor of NF-κB, no treatment control to determine the baseline activity of the NF-κB in OVCAR-3 cells, and no primary antibody control to test if there is any autofluorescence in the tested samples.

In this study, we detected the localization of NF-κB in the ovarian cancer OVCAR-3 cells as a measure of NF-κB activity. This method of detection is advantageous compared to other methods [28]. OVCAR-3 cells are known to have constitutively active NF-κB oncogenic pathway indicating poor clinical outcome [29], so we tested whether NSC745885 and NSC757963 can inhibit the constitutively active NF-κB pathway in ovarian cancer. We used the standard pharmacologic NF-κB inhibitor pyrrolidine dithiocarbamate (PDTC) as a positive treatment control [29]. In concordance with the literature, Fig 8 showed that the NF-κB p65 subunit is localized in the nucleus of non-treated OVCAR-3 cells indicating endogenous high activity of the NF-κB pathway. Both NSC745885 and NSC757963 were significantly capable of preventing the nuclear translocation of the NF-κB p65 subunit with subsequent accumulation of the NF-κB p65 subunit in the cytosol, similar to the effect observed with PDTC, indicating inhibition of the constitutively actived NF-κB pathway in the tested ovarian cancer cells. These results are supporting our COMPARE analysis results regarding the mechanism of action of our novel compounds as discussed previously in this article.

### NSC745885 and NSC757963 inhibit the constitutively active NF-κB pathway in a concentration dependent manner

**Fig 9:**
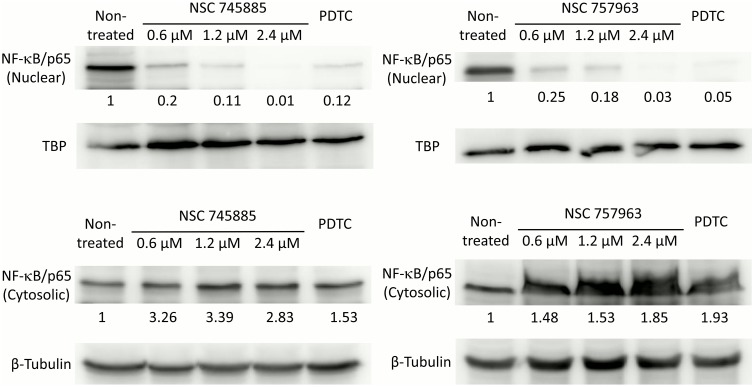
Western blotting of nuclear and cytosolic fractions of OVCAR-3 cells treated with different concentrations of either NSC745885, or NSC757963. Non-treated cells as well as the PDTC (50 μM) treated cells were used as negative and positive treatments controls.

To further confirm the inhibition of the constitutively active NF-κB pathway by our compounds, we performed Western blot experiments for the expression of NF-κB p65 subunit in nuclear and cytosolic extracts of OVCAR-3 cells (Fig 9). Non-treated cells showed high amount of the NF-κB p65 subunit in their nuclear extracts, confirming the high activation status of NF-κB of the tested cells. Treating the cells with NSC745885 significantly reduced the expression of NF-κB p65 subunit in the nuclear fraction of cells in a dose dependent manner with almost complete inhibition at a dose of 2.4 μM, subsequent increase of the NF-κB p65 subunit expression was observed in the cytosolic fractions of the treated cells. Similarly, NSC757963 treatment reduced significantly the expression of NF-κB p65 subunit in the nuclear fractions of the treated cells in a dose dependent manner with subsequent accumulation of the NF-κB p65 subunit in the cytosolic fractions, a dose of 2.4 μM could abrogate almost completely the nuclear translocation of NF-κB p65 subunit. These findings are in agreement with our results of the immunocytochemical imaging as well as the COMPARE analysis, and elucidate the inhibitory effects of our compounds on the constitutively active NF-κB pathway by impeding the translocation of NF-κB subunits to the nucleus. Such inhibitory effects have been observed with some drugs structurally related to our compounds like emodin [30, 31] and minocycline [29].

### Binding of NSC745885 and NSC757963 to IκB kinase & subsequent effect on the NF-κB pathway

IκB kinase (IKK) is a multiprotein complex that regulates activity of the NF-κB pathway by phosphorylating the inhibitory IκB proteins upon activation, leading to poly-ubiquitination and degradation of the inhibitory IκB proteins and subsequent activation and translocation of the NF-κB to the nucleus, to bind to its target DNA sequence and induce transcription of the target genes. IKK contains two kinase subunits IKKα and IKKβ, with IKKβ being more potent NF-κB activator owing to 20~50 fold higher kinase activity than IKKα. The crystal structure of the IKK subunits bound to the inhibitor K252a was published previously and available from the protein database website (code 4kik). The inhibitor K252a was found to bind at the ATP site of the N-terminal kinase domain of the IKK subunits, preventing phosphorylation of the IκB and subsequent inhibition of NF-κB activation and translocation to the nucleus [32].

**Fig 10:**
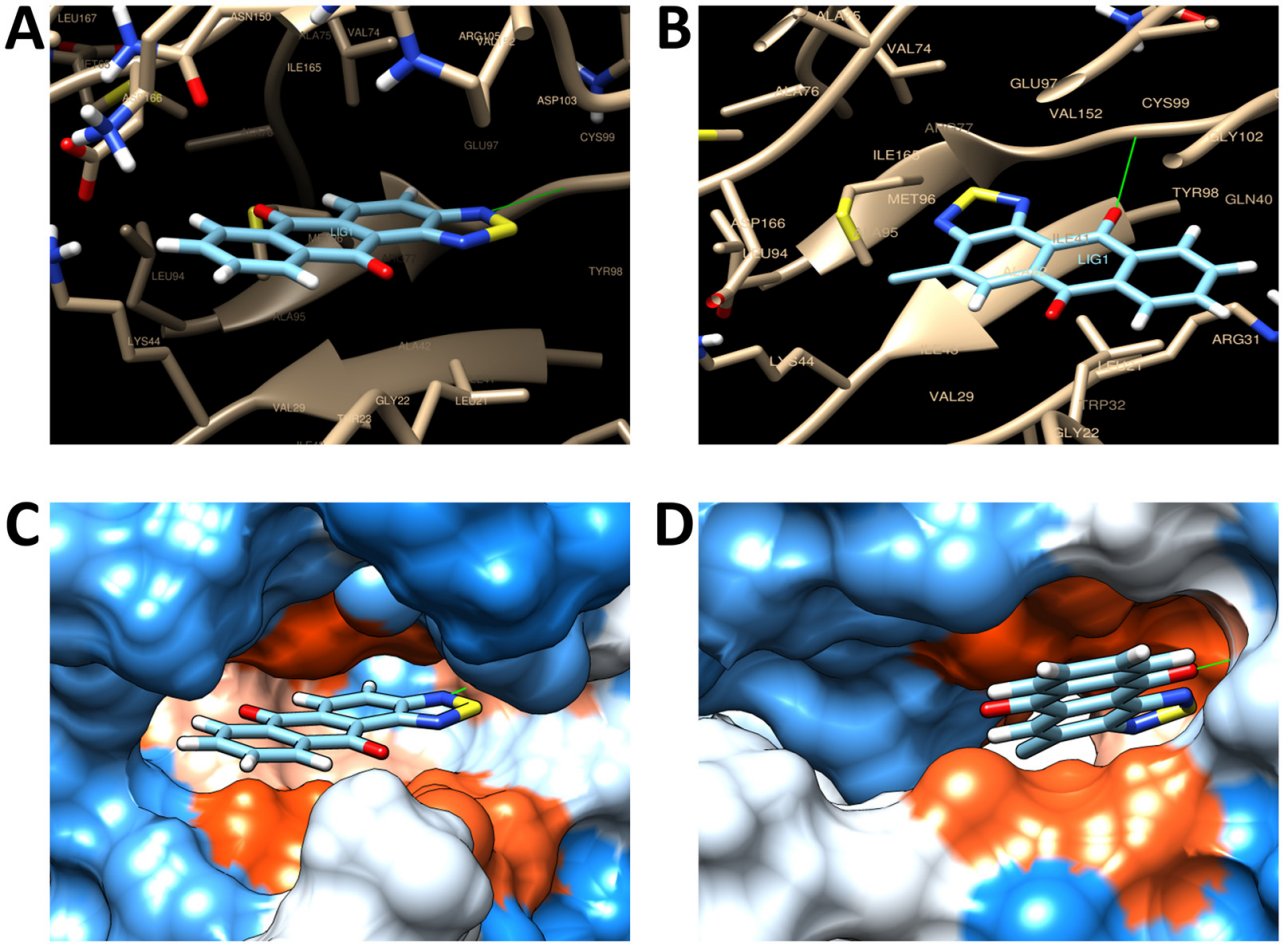
Binding poses of NSC745885 and NSC757963 into the ATP site of the N-terminal kinase domain of IKKβ. **A, B**: Visualization of the molecular interactions of NSC745885 **(A)** and NSC757963 **(B)** with the amino acids of the protein. Hydrogen bonding is presented as solid green lines. **C, D**: Binding of NSC745885 **(C)** and NSC757963 **(D)** to the hydrophobic pocket of the active site of the N-terminal kinase domain. Hydrophobic surfaces are presented in orange red colors, whereas hydrophilic surfaces are presented in blue colors.

In order to understand the mechanism of inhibition of the constitutively active NF-κB pathway by NSC745885 and NSC757963, as observed from the COMPARE analysis, immunocytochemical imaging and Western blot experiments, we tested the docking of both compounds into the IKKβ subunit using three molecular modeling software named SwissDock [33], PyRx using AutoDock Vina [34], and iGEMDOCK [35]. Results obtained from SwissDock showed favorable binding of both compounds to the ATP site of the N-terminal kinase domain of the IKKβ subunit, as shown in Fig 10, with Gibbs free energy of binding (ΔG) values of -7.57 kcal/mol for NSC745885 and -7.55 kcal/mol for NSC757963 for their most favorable binding modes. Careful examination of the binding poses of both compounds revealed hydrogen bonding between the CYS99 of the IKKβ kinase domain and the N and O atoms of NSC745885 and NSC757963 (presented as solid green lines in Fig 10A and 10B). Moreover, this binding site is located within a hydrophobic pocket walled with several hydrophobic amino acids such as ILE, VAL, LYS, ALA, CYS, and LEU, implying the existence of hydrophobic interactions between the binding site and both of our compounds, which stabilizes their binding to the active site (hydrophobic surfaces are colored as orange red in Fig 10C and 10D, whereas hydrophilic surfaces are colored as blue). Similar observations were obtained from PyRx results, where both compounds binded to the receptor with binding affinity values of -10.3 kcal/mol and -9.9 kcal/mol for NSC745885 and NSC757963, respectively. Along the same line, iGEMDOCK results showed similar binding modes for both compounds with Van der Waals energy values of -77.07 kcal/mol and -77.21 kcal/mol for NSC745885 and NSC757963, respectively, and hydrogen bonding energy values of -10.49 kcal/mol and -12.85 kcal/mol for NSC745885 and NSC757963, respectively. Collectively, these results indicate that NSC745885 and NSC757963 may bind to the N-terminal kinase domain of the IKKβ at its ATP site through hydrogen bonding and hydrophobic interactions, preventing it from phosphorylating the inhibitory IκB proteins, resulting in inhibition of the NF-κB translocation to the nucleus, accumulation of NF-κB in the cytosol, and subsequent suppression of the NF-κB transcriptional activity. These findings further support our COMPARE analysis, immunocytochemical imaging, and Western blot results of OVCAR-3 cells, where we observed significant inhibition of the cells’ constitutively active NF-κB, with cytosolic accumulation and suppressed nuclear expression of the NF-κB p65 subunit following treatment with our compounds. Similar findings have been observed with emodin, which has a core anthraquinone moiety in its chemical structure similar to our compounds. Emodin could effectively inhibit the degradation of IκB, translocation of the NF-κB to the nucleus and overall activity of the NF-κB pathway in different types of cells [30, 31].

### Activity of NSC757963 against *Mycobacterium tuberculosis*

The antimycobacterial activity of NSC757963 was tested against the standard reference strain of *Mycobacterium tuberculosis* H37Rv that is commonly used in evaluating the antimycobacterial activity of drugs [36, 37]. We used the BACTEC MGIT 960 SIRE system to determine the values of MIC, as this method proved to be highly stable, reproducible and accurate compared to other commonly used methods [36, 37]. Susceptibility of the H37Rv strain to control drugs displayed in Table 5 showed that the experimental procedures are held correctly and that the sensitivity of the tested strain is suitable for the antimycobacterial activity evaluation of our compound (Streptomycin, Isoniazid, Rifampin, and Ethambutol with MIC values 0.1 μg/mL~ 5 μg/mL).

**Table 5:** Susceptibility of *Mycobacterium tuberculosis* H37Rv towards NSC757963.

Tested Drugs	NSC757963							Streptomycin 1 μg/mL	Isoniazid 0.1 μg/mL	Rifampin 1 μg/mL	Ethambutol 5 μg/mL
	1.25 μg/mL	2.5 μg/mL	5 μg/mL	7.5 μg/mL	10 μg/mL	15 μg/mL	20 μg/mL				
**Effect on *M*. *tuberculosis* H37Rv**	Growth	Growth	Growth	Growth	Inhibition	Inhibition	Inhibition	Inhibition	Inhibition	Inhibition	Inhibition

NSC757963 showed a MIC of 10 μg/mL which is a potent low concentration that is not only similar to but also less than the MICs of some drugs used in clinical practice [7]. These results impose the importance of our compound to be developed as a novel antimycobacterial agent specially after the emergence of both multidrug-resistant tuberculosis (MDR-TB) and extensively drug-resistant tuberculosis (XDR-TB) [19, 20], and due to the fact that long term treatment with the currently used drugs is more likely to induce hepatotoxicity to the patients [18].

### Compliance of NSC745885 and NSC757963 to criteria of the prospective small molecular drugs

Physico-chemical properties of the novel lead compounds are very important to determine their suitability to proceed for further modifications until they approach clinical trials. For example, the logarithm values of the aqueous solubility measured in mol/liter (log S) determine the absorption and distribution properties of the drugs. Another important property is the total polar surface area (TPSA) which is the total sum of all polar regions of the molecule’s surface, this property correlates to the intestinal absorption, and blood brain barrier penetration as well as other bioavailability properties [38]. In 2001, Lipinski *et al*. studied the physico-chemical properties of more than 2000 drugs or drug candidates to correlate to the bioavailability properties, they determined some criteria for the compound to have good bioavailability and constructed the “Lipinski’s Rule of Five” [39]. A compound to have acceptable bioavailability, and thus to be considered as a possible drug candidate, should not violate more than one rule of the Lipinski’s Rule of Five [38]. This rule indicates that the molecular weight should be less than or equal to 500, computed octanol—water partition coefficient (CLogP) for lipophilicity should be less than or equal to 5, number of H-bond donors should be less than or equal to 5 and the number of H-bond acceptors should be less than or equal to 10 [40].

Results showed that NSC745885 and NSC757963 satisfy all criteria of the Lipinski’s rule of 5 as well as of the solubility, total polar surface area, and rotatable bonds (Table 6). This implies that both compounds may have acceptable bioavailability and ADME properties. Furthermore, both compounds did not show any predicted mutagenicity, tumorigenicity, irritability or reproductive effects.

**Table 6:** Molecular and ADME-related properties of NSC745885 and NSC757963.

Property	NSC745885	NSC757963	Acceptable limits
LogS (Solubility)	-1.36	-2.1	≥—4
CLogP (Lipophilicity)	3.55	4.16	≤ 5
Molecular Weight	266.0146	299.9766	≤ 500
Total polar surface area (Å)	88.16	88.16	< 100
Number of hydrogen bond acceptors	4	4	≤ 10
Number of hydrogen bond donors	0	0	≤ 5
Violations to Lipinski’s rule	0	0	1
Rotatable bonds	0	0	< 10

### Determinig the oral absorption of NSC745885 through the human intestinal cells

We used the Caco-2 permeability experiments as a well-established model of human intestinal absorption of drugs [9]. Human Caco-2 cells have the advantages of differentiating into fully polarized cells with well-established tight junctions and brush border membrane structures. Furthermore, these cells exhibit the metabolizing enzymes as well as the membrane transport proteins, efflux proteins and Phase II conjugation enzymes that determine the metabolism and transport of drugs by a variety of transcellular pathways across the intestines (passive diffusion, carrier-mediated, and vesicular transport) [41, 42].

We tested both the absorptive (from the apical to basolateral compartments; A→BL) and secretory (from the basolateral to apical compartments; BL→A) permeabilities of our compound. We determined the amounts of compound transported at the specified time points and calculated the cumulative fractions [F _(cum)_] transported with respect to time according to the following equation [43]:

$$F_{\left(cum\right)}=\sum^{\text{​}}\frac{\left[C_{R}\left(t_{k}\right)-fC_{R}\left(t_{k-1}\right)\right].V_{R}}{C_{D}.V_{D}}$$

Where C is the concentration (μM), V is the volume (mL) of the receiver (R) or donor (D), t_k_ is time point k, and *f* is the sample replacement factor calculated as $f=\left(1-\frac{V_{s}}{V_{R}}\right)$, where V_S_ is the volume of the sample (mL).

We determined the permeability coefficients as follows:

$$P_{app}=\left(\frac{\Delta F_{\left(cum\right)}}{\Delta t}\right).\left(\frac{1}{A}\right).V_{D}$$

Where $\left(\frac{\Delta F_{\left(cum\right)}}{\Delta t}\right)$ is the slope of the graph of F_(cum)_ versus time (s), A is the area of the filter (cm^2^), and V_D_ is the volume of the donor compartment (mL).

We determined the active transport by calculating the uptake ratios and the intestinal efflux by calculating the efflux ratios using the following equations [8]:

$$Uptake\;\;ratio=\frac{P_{app\left(A\to BL\right)}}{P_{app\left(BL\to A\right)}}$$

$$Efflux\;\;ratio=\frac{P_{app\left(BL\to A\right)}}{P_{app\left(A\to BL\right)}}$$

Where *P*_*app(A→BL)*_ is the absorptive permeability coefficient, and *P*_*app(BL→A)*_ is the secretory permeability coefficient.

**Fig 11:**
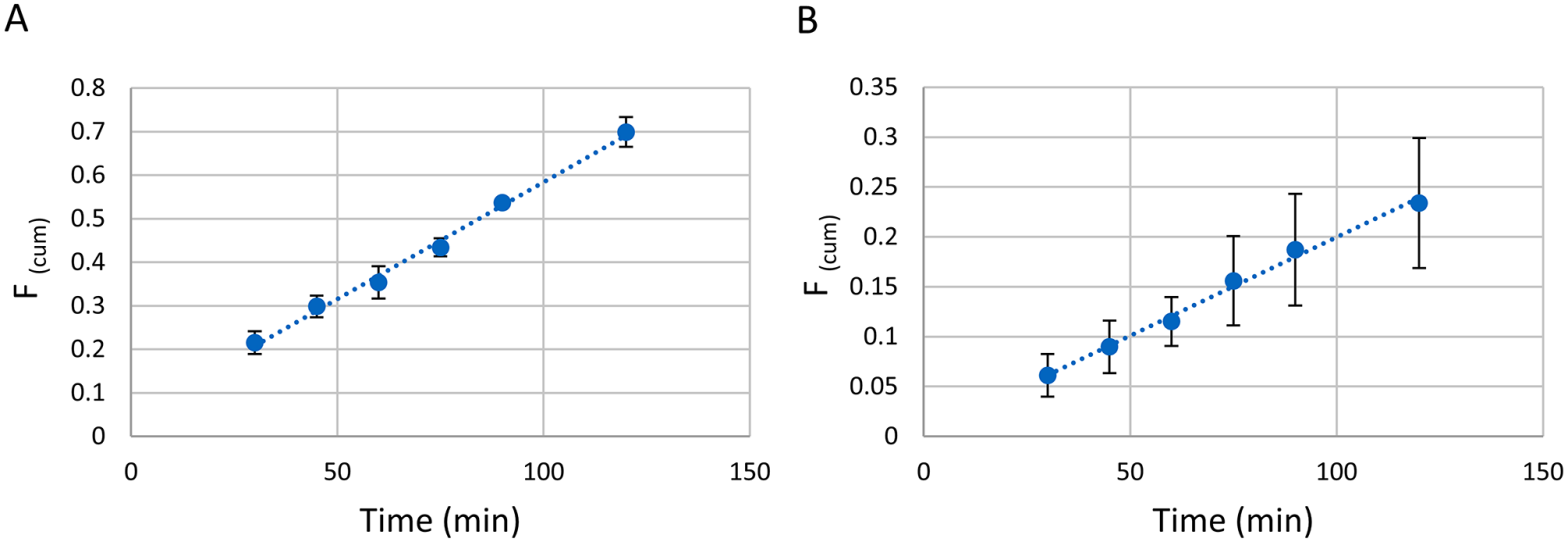
Cumulative fractions of NSC745885 transported in the absorptive transport direction (A) and in the secretory transport direction (B) across the human intestinal Caco-2 cells with respect to time.

We used low enough concentrations of NSC745885 to avoid toxicity to the CaCo-2 cells (did not compromise the cell integrity which was confirmed by measuring the transepithelial electric resistance (TEER) across the cell monolayers at the end of experiments [44]). Also, such low concentrations were aimed to avoid saturation of the transporter proteins involved in the active transport and efflux processes by the test compound [8]. The cumulative fractions of NSC745885 transported in both directions across the Caco-2 monolayers with respect to time are presented in Fig 11. It is reported that drugs with permeability coefficients more than 1x10^-6^ cm/sec are completely absorbed from the human gastrointestinal tract when administered orally [45]. Results presented in Table 7 showed that NSC745885 has high absorptive permeability coefficient (31.8x10^-6^ cm/sec) as well as a high secretory permeability coefficient (35.4x10^-6^ cm/sec) indicating that NSC745885 is completely absorbed in humans when taken orally. The uptake ratio was found to be 0.898 indicating absence of active transport for the absorption of NSC745885, while the efflux ratio was 1.11 indicating absence of intestinal efflux to NSC745885 according to Hubatsch *et*. *al*., 2007 [8]. This led us to identify the mechanism of absorption of NSC745885 to be only passive diffusion without involvement of transporter proteins. The absence of efflux effects to NSC745885, observed from our results, is of special importance because intestinal efflux of drugs usually results in poor oral bioavailability as the absorbed drugs are secreted back into the intestinal lumen.

**Table 7:** Intestinal absorption and secretion of NSC745885 obtained from the Caco-2 permeability experiments*.

Absorptive permeability P_app(A→BL)_ (cm/sec)	Secretory permeability P_app(BL→A)_ (cm/sec)	Uptake ratio	Efflux ratio	Permeability ranking
31.8x10^-6^	35.4x10^-6^	0.898	1.11	High; completely absorbed

* The values presented in this table are obtained from three independent experiments.

## Materials and Methods

### Chemical synthesis of NSC745885 and NSC757963

For NSC745885, 1,2-diaminoanthraquinone (1.19 g, 5 mmol) dissolved in tetrahydrofuran (30 mL) was reacted with thionyl chloride (0.5 mL) and triethylamine (3 mL) for 1 h at room temperature. The reaction mixture was treated with crushed ice (100 mL) and the resulting precipitate was washed with diethyl ether and purified by crystallization from ethanol [4]. NSC745885 was obtained as a yellowish powder (yield 74%). Purity determined by HPLC was 98.06% with a retention time of 3.97 min (S6 Fig). ^1^H NMR (300 MHz, CDCl_3_) spectrum is illustrated in S7 Fig. For NSC757963, a solution of NSC745885 (0.5 g, 2 mmol) in glacial acetic acid (15 mL) was treated with HCl (5 mL) at 90°C, potassium chlorate (2 g) was added to the reaction mixture over a period of 45 min then the reaction mixture was kept boiling for 4 h. The product was then precipitated by cooling in an ice bath and crystallized from acetic acid, synthesis steps are summarized in S8 Fig [46]. NSC757963 was obtained as a yellowish orange powder (yield = 72%). Purity determined by HPLC was 95.96% with a retention time of 4.16 min (S9 Fig). Mp: 253–254°C (EtOH). ^1^H NMR (300 MHz, CDCl_3_): *δ* ppm 7.84–7.9 (2H, m, Ar-H_8_, Ar-H_9_), 8.31(1H, dd, *J* = 9.0 Hz, *J* = 1.5 Hz, Ar-H_10_), 8.38(1H,dd, *J* = 9.0 Hz, *J* = 1.9 Hz, Ar-H_7_), 8.61 (1H, s, Ar-H_5_), its spectrum is illustrated in S10 Fig. ^13^C NMR (75 MHz, CDCl_3_): *δ* ppm 123.83, 124.88, 126.62, 130.87, 131.60, 133.16, 134.43, 135.11, 135.48, 151.06, 154.70, 180.70, 182.10. HRMS (EI) m/z:calcd [M]+, 299.9760 (C14H5ClN2O2S2+); found, 299.9766.

### Cytotoxic activity evaluation of our synthesized compounds

The cell lines used in this study were obtained by the NCI and JFCR as well as from the Food Industry Research and Development Institute, Taiwan. All cell lines were published before [4, 22, 47]. The cytotoxicity screening experiments were performed according to our previous publication [4]. Briefly, 96 well microtiter plates were inoculated with the specified human cancer cell lines in RPMI 1640 medium containing 5% fetal bovine serum and 2 mM L-glutamine at the specified plating densities. The microtiter plates were incubated at 37°C, 5% CO_2_, and 100% relative humidity for 24 h. The test compounds were solubilized in DMSO at 400-fold the desired final maximum test concentration, diluted with complete medium containing gentamicin and added to the wells of the microtiter plates to achieve the final desired compound concentration, then incubated for an additional 48 h at the same incubation conditions. The media were removed, the cells were fixed then stained with sulforhodamine B and the unbound dye was removed by washing with acetic acid. The bound dye was resolubilized with trizma base and the absorbance at 515 nm was measured. Cells were stained with the WST-1 cell proliferation reagent instead of sulforhodamine B in some experiments and absorbance were measured at 440 nm. The three dose response parameters GI_50_, TGI and LC_50_ were calculated for each experimental compound as follows [48]:

For values where Ti ≥ Tz the formula is [ (Ti-Tz) / (C-Tz)] x 100

For values where Ti < Tz the formula is [ (Ti-Tz) / Tz] x 100

Where **Ti** is the absorbance of the solubilized Sulforhodamine B (SRB) (representing the number of cells) of the five different concentrations of the compound used in the test, **Tz** is the absorbance of the SRB at time zero, and **C** is the absorbance of the SRB of the control growth.

### Identifying the potential molecular targets and the therapeutic classes of NSC745885 and NSC757963

The activity patterns of both NSC745885 and NSC757963 (fingerprints) were used as “seed” in the COMPARE algorithms to correlate to those in the NCI databases using the Pearson’s correlation coefficient method. Briefly, the mean graph values of test compounds were correlated to those of the “Standard Agents” and “Marketed Drugs” databases as well as to thousands of molecular targets (determined using the protein levels, RNA measurements, mutation status and enzyme activity levels of the used cell lines) using the Structured Query language (SQL) and SAS programs [49]. Similar methods were performed to obtain the correlation results from the JFCR databases.

### Testing the NF-κB inhibition by immunocytochemical fluorescent staining

Inhibition of NF-κB was detected according to Ataie-Kachoie *et al*. [29]. Briefly, each group of tested cells were seeded on coverslips treated with HCl and ethanol, and autoclaved prior to use. Immunostaining of the p65 subunit of NF-κB was done by permeabilizing the cells with Triton X-10, then by treating the cells with anti-NF-κB p65 rabbit monoclonal primary antibody [E379] Abcam # ab32536, followed by Alexa Fluor^®^ 488 Donkey anti-rabbit IgG secondary antibody Biolegend # 406416. Nuclei of cells were stained with 4',6-diamidino-2-phenylindole (DAPI). Images were acquired using fluorescence microscope. Tested cells were divided into five groups which are no treatment control, no primary antibody control, PDTC (50 μM) treated control, NSC745885 (5 μM) and NSC757963 (5 μM) treated controls.

### Molecular docking

We downloaded the crystal structure of IKK protein complex from the RCSB protein databank website http://www.rcsb.org/pdb/home/home.do by searching for the code 4KIK (only the IKKβ subunit was used in docking). We determined the binding site with a 10 Angstrom sphere around the cocrystallized inhibitor K252a using the Swiss-PdbViewer DeepView software [50]. For docking using SwissDock [33], we used accurate mode with flexibility of side chains to determine the best docking poses of the compounds into the protein [51] and visualized the results using UCSF Chimera software [52]. For docking using PyRx [34], we used the AutoDock Vina wizard. For docking using iGEMDOCK [35], we used the “Stable Docking” as the default setting.

### Western blotting for protein expression

Treated cells were fractionated into nuclear and cytosolic fractions using the Nuclear/Cytosol fractionation kit (BioVision), separated by electrophoresis in SDS-PAGE gels and transferred to Amersham Hybond-P PVDF Membranes. Membranes were probed with either anti-NF-κB p65 rabbit monoclonal primary antibody [E379] Abcam # ab32536, anti-TATA binding protein (TBP) mouse antibody (1TBP18), or anti-beta Tubulin rabbit antibody [NB600-936]. After washing, membranes were incubated with either goat anti-rabbit IgG H&L (HRP) (ab6721), or rabbit anti-mouse IgG H&L (HRP) (ab6728) secondary antibodies. Membranes were developed using the Immobilon Western chemiluminescent HRP substrate kit. Protein bands were normalized to either the beta Tubulin or TBP bands and quantified using ImageJ software.

### Testing the antimycobacterial susceptibility of *Mycobacterium tuberculosis* to NSC757963

The antimycobacterial susceptibility of *Mycobacterium tuberculosis* to NSC757963 was tested using the BACTEC ^™^ MGIT ^™^ 960 SIRE Kit [53]. Briefly, *Mycobacterium tuberculosis* H37Rv reference strain was cultured in 96 well plates containing Middlebrook 7H9 broth with or without the compounds to be tested at different concentrations (20, 15, 10, 7.5, 5, 2.5, 1.25 μg/mL). Cultures were incubated for 14 days after which growth in every well was observed. Susceptibility to the tested drugs was calculated as the minimum concentration of the drug that can completely inhibit the growth of *M*. *tuberculosis*. Four antimycobacterial drugs, which are Streptomycin (1 μg/mL), Isoniazid (0.1 μg/mL), Rifampin (1 μg/mL), and Ethambutol (5 μg/mL) were tested along with NSC757963 as control drugs to assure the susceptibility of the tested strain and to validate the experimental procedures.

### Determining the purity and concentrations of compounds using the HPLC

Chromatographic separations and analysis were performed using a Hitachi L-2000 series system with UV detector (L-2400, Hitachi) equipped with a C18 reverse-phase column (XBridge BEH Shield RP18 Column, 130 Å, 5 μm, 4.6 mm × 250 mm, Waters). Analyses for NSC745885 and NSC757963 were performed using their maximum absorbance wavelengths (260 nm and 262 nm, respectively). HPLC grade methanol was used as the mobile phase and ran at a rate of 1.0 mL/min.

### Determinig the molecular properties and applying the Lipinski’s rule of five

The properties displayed in Table 6 are calculated using the Osiris Property Explorer [31], accessed March 4, 2014, and the toxicity indications were based on statistically derived fragment lists [54].

### Determinig the absorption of compounds through the human intestinal Caco-2 cells

The transport of our compounds through the Caco-2 cells was tested according to Hubatsch et al., 2007 [8]. Briefly, Caco-2 cells were seeded at a density of 2.6 × 10^5^ cells/cm^2^ onto Corning Transwell^®^ polyester membrane inserts (1.2 cm^2^ membrane diameter, 0.4 μm pore size, product #3460) in 12-well tissue culture plates and cultured in Dulbecco’s modified Eagle’s medium with high glucose (4500 mg/liter glucose) and L-glutamine (without pyruvate) supplemented with 10% FBS, 1% non-essential amino acids and 1% penicillin/streptomycin. Cells were incubated for at least 21 days at the specified conditions with the change of medium every two days. The TEER was measured using Millicell^®^ ERS device for each insert after every change of medium to monitor the development of the cells monolayer. Cell monolayers with TEER values less than 300 Ω.cm^2^ at the end of the maintenance period were not suitable for the experiment and discarded [43]. The integrity of the cell monolayers were further confirmed by testing the phenol red permeability through the cells [55]. The transport experiments were started by replacing the incubation culture media with the transport buffers with or without the tested compounds, the transport was tested in both directions (apical to basolateral and basolateral to apical). We used Hank’s balanced salt solution without phenol red as the transport medium buffered with either methanesulfonic acid (pH = 6.5) as the transport buffer in the apical compartment or buffered with HEPES and NaHCO_3_ (pH = 7.4) as the transport buffer in the basolateral compartment. NSC745885 was dissolved in the transport buffers with the aid of 1% DMSO to get final concentrations between 4–5 μM. Samples from the receiver compartments were withdrawn at different time intervals (0 min, 30 min, 45 min, 60 min, 75 min, 90 min, 120 min) and the amounts of transported drug were determined using the HPLC. After the transport experiments, the TEER was checked for every cell monolayer to assure that the monolayers were not compromised from the effect of the tested drug. The apparent permeabilities were calculated as mentioned in the previous section.

## Conclusions

Based on the cytotoxic spectra of activity of NSC745885 and NSC757963, their correlations to compounds of the NCI and the JFCR databases, and the identified molecular targets, we present two promising selective lead compounds with wide spectrum of cytotoxic activities against different cancer types rather than normal cells, and acceptable molecular, bioavailability and ADME properties. Both compounds exhibit activity spectra comparable to those of the standard drugs of the NCI database that are currently used in the clinical practice or in the clinical trials, with NSC757963 displaying high selectivity towards the leukemia subpanel. Both compounds have strong correlations with many effective molecular targets that are involved in the anticancer treatment strategies such as inhibiting the NF-κB which was confirmed by the immunocytochemical imaging, Western blotting and molecular docking. Moreover, activities of both compounds were not correlated to the expression of MGMT chemoresistance gene. Furthermore, NSC757963 may present a novel anti-tuberculosis agent, this was confirmed by determining the MIC against the *Mycobacterium tuberculosis* H37Rv strain. Finally, results showed that NSC745885 will be completely absorbed from the human intestine if administered orally. In short, the findings of this study provide an integral part of the pre-clinical investigation of our lead compounds that warrant their further development as anticancer agents against the multi-drug resistant tumors with excellent oral absorption.

## Supporting Information

S1 Fig Mean Graph of the log_10_ values (Molar) of GI_50_, TGI and LC_50_ of NSC745885 obtained from the NCI 60 cell line experiments. X-axis is constructed based on the log_10_ scale, the zero represents log_10_ of the mean values (MID or MG-MID) of each of the GI_50_, TGI and LC_50_. Values to the right side of zero indicate more sensitivity of the cell lines to the tested compound than the mean value and those to the left side indicate more resistance to the tested compound than the mean value. Delta values are the difference between the mean values (MID or MG-MID) and the log_10_ of each corresponding values of the GI_50_, TGI and LC_50_ for the most sensitive cell line. Range values are the difference between log_10_ each of the GI_50_, TGI and LC_50_ values for the most resistant cell line and log_10_ each of the corresponding values of GI_50_, TGI and LC_50_ for the most sensitive cell line.
(TIF)

S2 Fig Mean Graph of the log_10_ values (Molar) of GI_50_, TGI and LC_50_ of NSC745885 obtained from the JFCR 39 cell line experiments. X-axis is constructed based on the log_10_ scale; log_10_ of the mean values (MG-MID) of each of GI_50_, TGI and LC_50_ are represented by the zero on the X-axis. Delta values are the difference between the MG-MID and the log_10_ of each corresponding values of the GI_50_, TGI and LC_50_ for the most sensitive cell line. Range values are the difference between log_10_ each of the GI_50_, TGI and LC_50_ values for the most resistant cell line and log_10_ each of the corresponding values of GI_50_, TGI and LC_50_ for the most sensitive cell line. Values to the right side of zero indicate more sensitivity of the cell lines to the tested compound than the mean and those to the left side indicate more resistance to the tested compound than the mean. Br: breast, CNS: central nervous system, Co: colon, Lu: lung, Me: melanoma, Ov: ovary, Re: renal, St: stomach, xPg: prostate.
(TIF)

S3 Fig Mean Graph of the log_10_ values (Molar) of GI_50_, TGI and LC_50_ of NSC757963 obtained from the NCI 60 cell line experiments. X-axis is constructed based on the log_10_ scale, the zero represents log_10_ of the mean values (MID or MG-MID) of each of the GI_50_, TGI and LC_50_. Values to the right side of zero indicate more sensitivity of the cell lines to the tested compound than the mean value and those to the left side indicate more resistance to the tested compound than the mean value. Delta values are the difference between the mean values (MID or MG-MID) and the log_10_ of each corresponding values of the GI_50_, TGI and LC_50_ for the most sensitive cell line. Range values are the difference between log_10_ each of the GI_50_, TGI and LC_50_ values for the most resistant cell line and log_10_ each of the corresponding values of GI_50_, TGI and LC_50_ for the most sensitive cell line.
(TIF)

S4 Fig Mean Graph of the log_10_ values (Molar) of GI_50_, TGI and LC_50_ of NSC757963 obtained from the JFCR 39 cell line experiments. X-axis is constructed based on the log_10_ scale; log_10_ of the mean values (MG-MID) of each of GI_50_, TGI and LC_50_ are represented by the zero on the X-axis. Delta values are the difference between the MG-MID and the log_10_ of each corresponding values of the GI_50_, TGI and LC_50_ for the most sensitive cell line. Range values are the difference between log_10_ each of the GI_50_, TGI and LC_50_ values for the most resistant cell line and log_10_ each of the corresponding values of GI_50_, TGI and LC_50_ for the most sensitive cell line. Values to the right side of zero indicate more sensitivity of the cell lines to the tested compound than the mean and those to the left side indicate more resistance to the tested compound than the mean. Br: breast, CNS: central nervous system, Co: colon, Lu: lung, Me: melanoma, Ov: ovary, Re: renal, St: stomach, xPg: prostate.
(TIF)

S5 Fig Cytotoxicity of NSC745885, NSC757963 and doxorubicin towards the normal cardiac myoblast H9c2 cells. (TIF)

S6 Fig HPLC chromatogram of NSC745885 showing purity of 98.06%. (TIF)

S7 Fig ^1^H-NMR spectrum of NSC745885. (TIF)

S8 Fig Synthesis scheme of NSC745885 and NSC757963. (TIF)

S9 Fig HPLC chromatogram of NSC757963 showing purity of 95.96%. (TIF)

S10 Fig ^1^H-NMR spectrum of NSC757963. (TIF)

S1 Table Mean GI_50_ values (Molar) and selectivity ratios of NSC745885 and NSC757963 obtained from the NCI 60 cell line experiments. ^a^ The average value of GI_50_ of every cell line panel tested in the five-dose NCI 60 cell line screen experiments. ^b^ The average value of GI_50_ of all of the tested cell lines in the five-dose NCI 60 cell line screen experiments and is equivalent to the mean graph midpoint (MID). ^c^ Compounds are rated as “selective” to the cell line panel if the ratio is more than 6, rated as “moderately selective” if the ratio is between 3 and 6, and rated as “non selective” if the ratio is less than 3.
(DOCX)

S2 Table Mean GI_50_ values (Molar) and selectivity ratios of NSC745885 and NSC757963 obtained from the JFCR 39 cell line experiments. ^a^ The average value of GI_50_ of every cell line panel tested in the five-dose JFCR 39 cell line screen experiments. ^b^ The average value of GI_50_ of all of the tested cell lines in the five-dose JFCR 39 cell line screen experiments and is equivalent to the mean graph midpoint (MG-MID). ^c^ Compounds are rated as “selective” to the cell line panel if the ratio is more than 6, rated as “moderately selective” if the ratio is between 3 and 6, and rated as “non selective” if the ratio is less than 3.
(DOCX)

S3 Table NCI STANDARD AGENTS with similar activity profiles to NSC745885. ^a^ Only compounds showing salient correlations with NSC745885 were selected and displayed in this table. Compounds appearing more than once in the COMPARE analysis results (due to difference in number of tested cell lines or in the hiConc of the compared experiments) were not included in this table. However the order of ranking of all of the compared compounds is retained. ^b^ This coefficient ranges from -1 to +1. Compounds with positive coefficient values approaching 1 have high similarities with the test compound, while those with negative coefficient values approaching -1 have high differences with the test compound.
(DOCX)

S4 Table NCI MARKETED DRUGS with similar activity profiles to NSC745885. ^a^ Only compounds showing salient correlations with NSC745885 were selected and displayed in this table. Compounds appearing more than once in the COMPARE analysis results (due to difference in number of tested cell lines or in the hiConc of the compared experiments) were not included in this table. However the order of ranking of all of the compared compounds is retained. ^b^ This coefficient ranges from -1 to +1. Compounds with positive coefficient values approaching 1 have high similarities with the test compound, while those with negative coefficient values approaching -1 have high differences with the test compound.
(DOCX)

S5 Table JFCR drugs with similar activity profiles to NSC745885. ^a^ This coefficient ranges from -1 to +1. Compounds with positive coefficient values approaching 1 have high similarities with the test compound, while those with negative coefficient values approaching -1 have high differences with the test compound.
(DOCX)

S6 Table NCI STANDARD AGENTS with similar activity profiles to NSC757963. ^a^ Only compounds showing salient correlations with NSC757963 were selected and displayed in this table. Compounds appearing more than once in the COMPARE analysis results (due to difference in number of tested cell lines or in the hiConc of the compared experiments) were not included in this table. However the order of ranking of all of the compared compounds is retained. ^b^ This coefficient ranges from -1 to +1. Compounds with positive coefficient values approaching 1 have high similarities with the test compound, while those with negative coefficient values approaching -1 have high differences with the test compound, and a value of zero indicates no correlation at all.
(DOCX)

S7 Table JFCR drugs with similar activity profiles to NSC757963. ^a^ This coefficient ranges from -1 to +1. Compounds with positive coefficient values approaching 1 have high similarities with the test compound, while those with negative coefficient values approaching -1 have high differences with the test compound, and a value of zero indicates no correlation at all.
(DOCX)
