# Supplementary figures and images for: Novel Anthra[1,2-c][1,2,5]Thiadiazole-6,11-Diones as Promising Anticancer Lead Compounds: Biological Evaluation, Characterization & Molecular Targets Determination
Source: PLoS One. 2016 Apr 21;11(4):e0154278. doi: 10.1371/journal.pone.0154278 (PMC4839570; doi:10.1371/journal.pone.0154278)

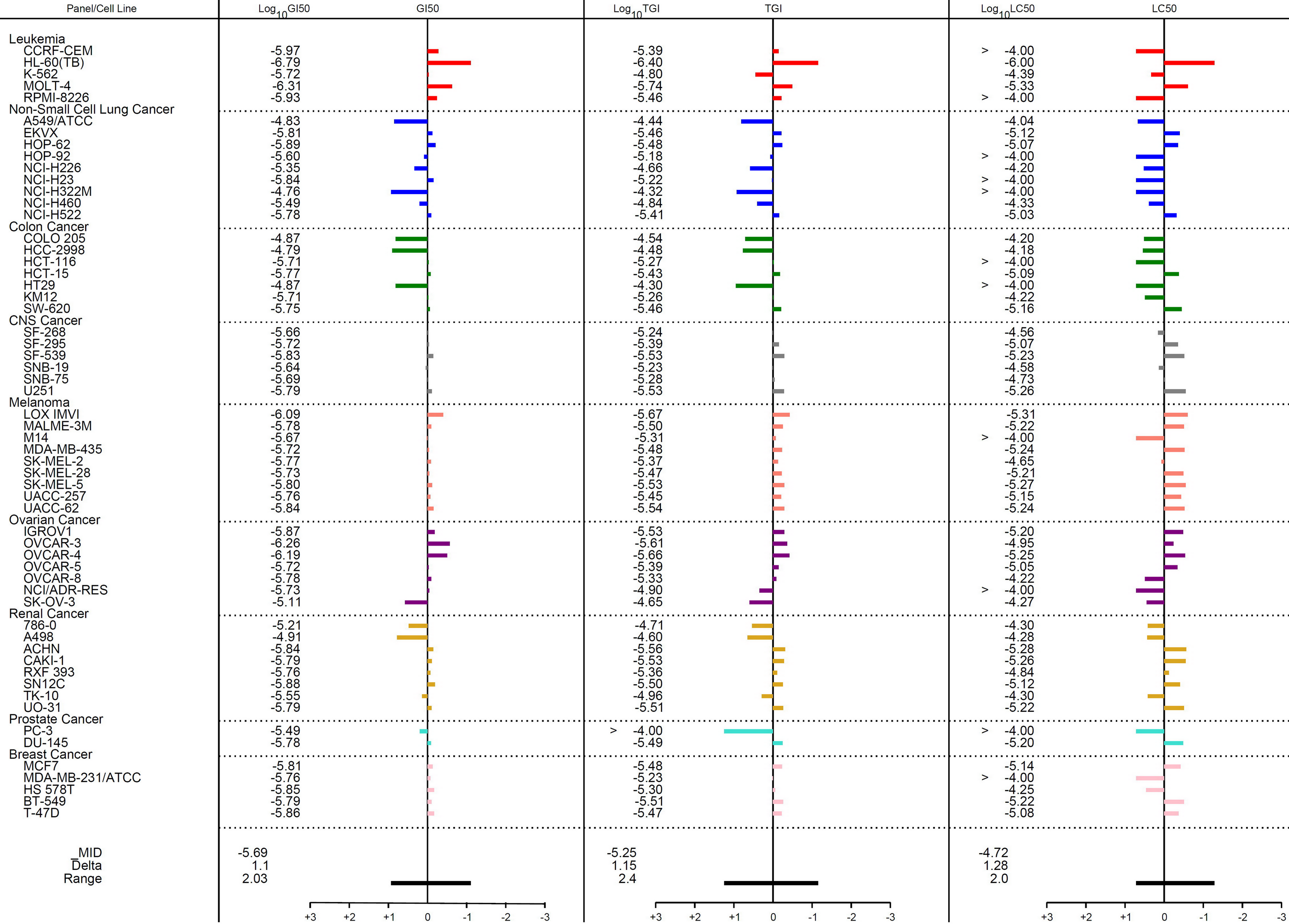

Supplement: S1 Fig — X-axis is constructed based on the log10 scale, the zero represents log10 of the mean values (MID or MG-MID) of each of the GI50, TGI and LC50. Values to the right side of zero indicate more sensitivity of the cell lines to the tested compound than the mean value and those to the left side indicate more resistance to the tested compound than the mean value. Delta values are the difference between the mean values (MID or MG-MID) and the log10 of each corresponding values of the GI50, TGI and LC50 for the most sensitive cell line. Range values are the difference between log10 each of the GI50, TGI and LC50 values for the most resistant cell line and log10 each of the corresponding values of GI50, TGI and LC50 for the most sensitive cell line. (TIF) [file pone.0154278.s001.tif]

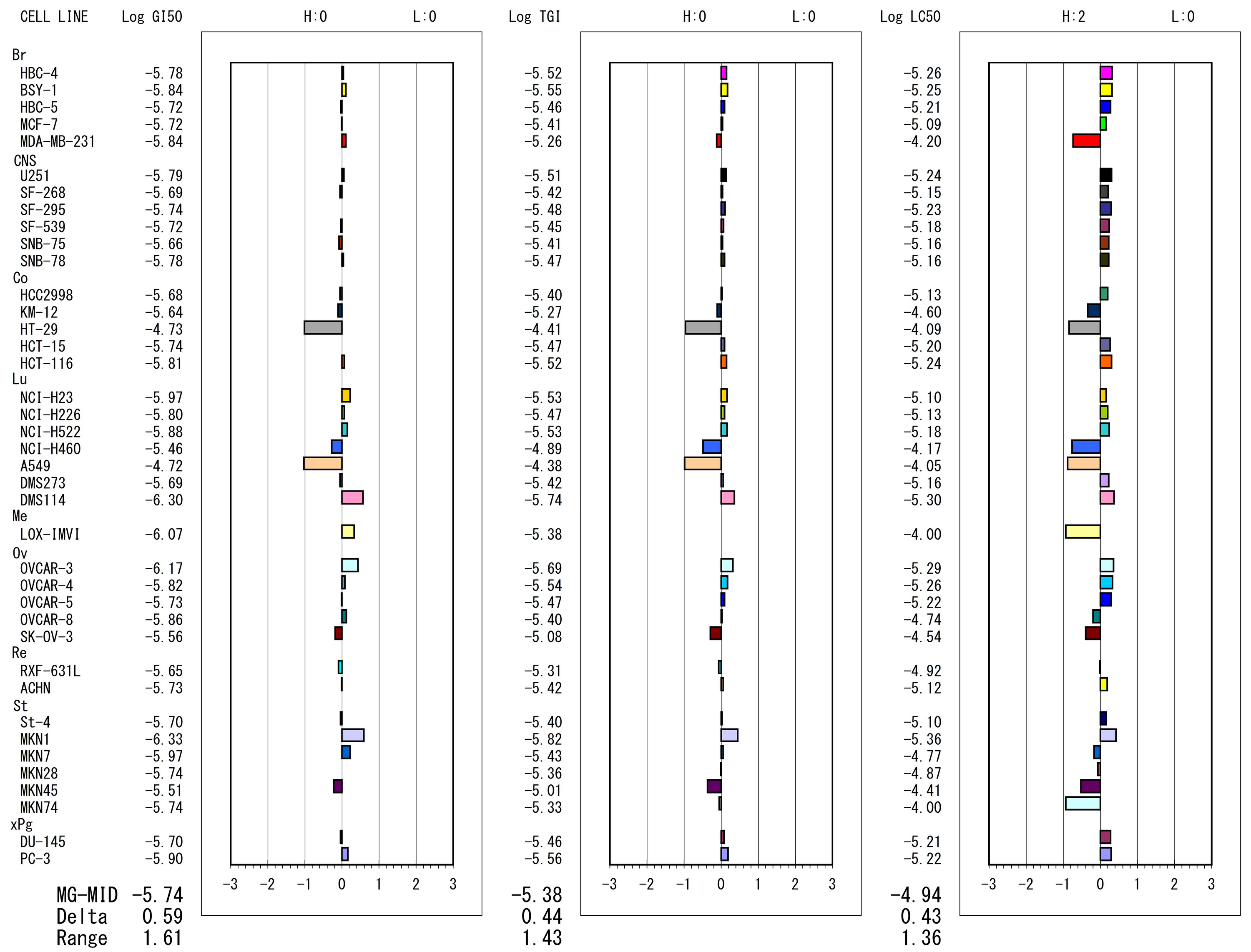

Supplement: S2 Fig — X-axis is constructed based on the log10 scale; log10 of the mean values (MG-MID) of each of GI50, TGI and LC50 are represented by the zero on the X-axis. Delta values are the difference between the MG-MID and the log10 of each corresponding values of the GI50, TGI and LC50 for the most sensitive cell line. Range values are the difference between log10 each of the GI50, TGI and LC50 values for the most resistant cell line and log10 each of the corresponding values of GI50, TGI and LC50 for the most sensitive cell line. Values to the right side of zero indicate more sensitivity of the cell lines to the tested compound than the mean and those to the left side indicate more resistance to the tested compound than the mean. Br: breast, CNS: central nervous system, Co: colon, Lu: lung, Me: melanoma, Ov: ovary, Re: renal, St: stomach, xPg: prostate. (TIF) [file pone.0154278.s002.tif]

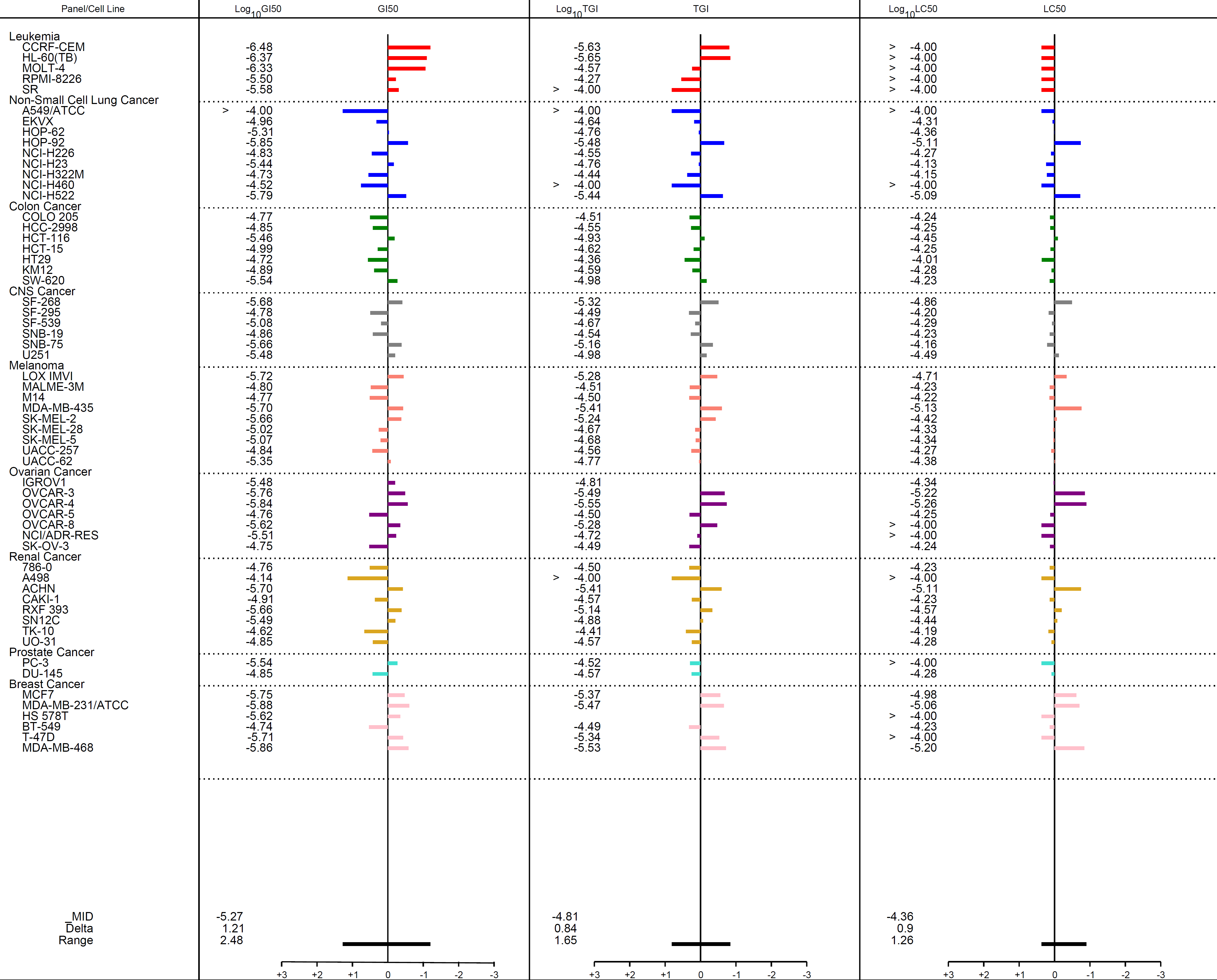

Supplement: S3 Fig — X-axis is constructed based on the log10 scale, the zero represents log10 of the mean values (MID or MG-MID) of each of the GI50, TGI and LC50. Values to the right side of zero indicate more sensitivity of the cell lines to the tested compound than the mean value and those to the left side indicate more resistance to the tested compound than the mean value. Delta values are the difference between the mean values (MID or MG-MID) and the log10 of each corresponding values of the GI50, TGI and LC50 for the most sensitive cell line. Range values are the difference between log10 each of the GI50, TGI and LC50 values for the most resistant cell line and log10 each of the corresponding values of GI50, TGI and LC50 for the most sensitive cell line. (TIF) [file pone.0154278.s003.tif]

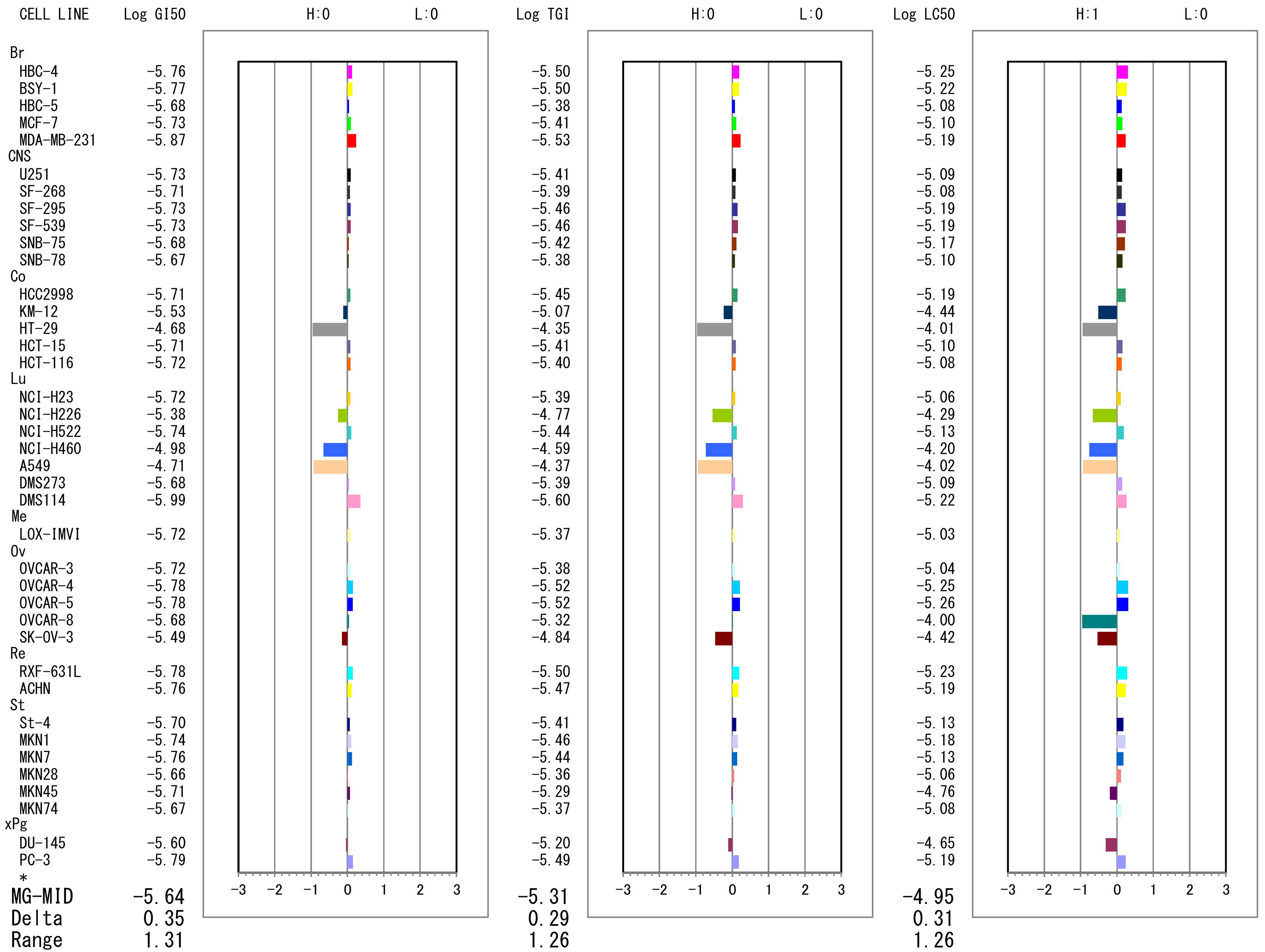

Supplement: S4 Fig — X-axis is constructed based on the log10 scale; log10 of the mean values (MG-MID) of each of GI50, TGI and LC50 are represented by the zero on the X-axis. Delta values are the difference between the MG-MID and the log10 of each corresponding values of the GI50, TGI and LC50 for the most sensitive cell line. Range values are the difference between log10 each of the GI50, TGI and LC50 values for the most resistant cell line and log10 each of the corresponding values of GI50, TGI and LC50 for the most sensitive cell line. Values to the right side of zero indicate more sensitivity of the cell lines to the tested compound than the mean and those to the left side indicate more resistance to the tested compound than the mean. Br: breast, CNS: central nervous system, Co: colon, Lu: lung, Me: melanoma, Ov: ovary, Re: renal, St: stomach, xPg: prostate. (TIF) [file pone.0154278.s004.tif]

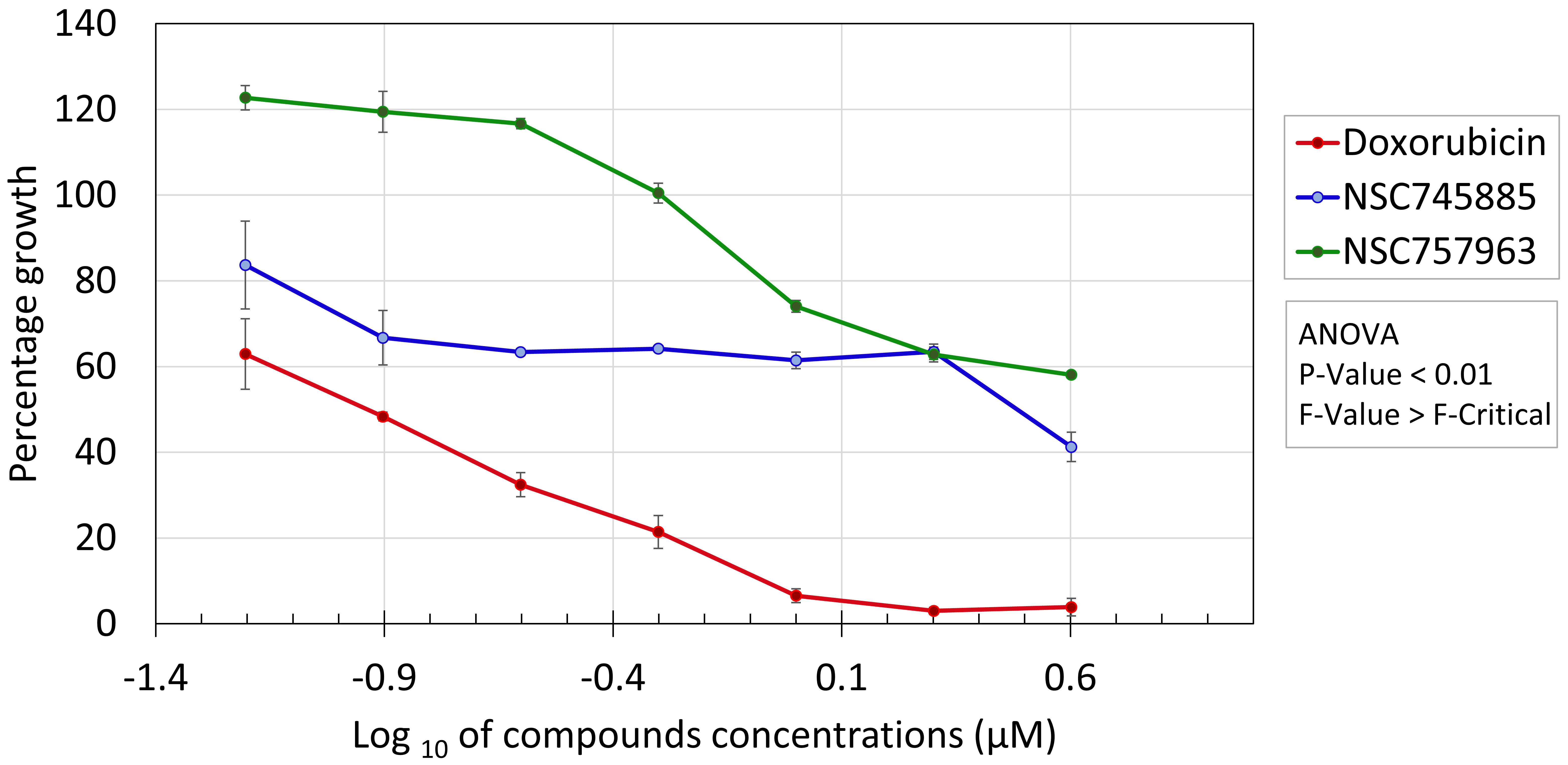

Supplement: S5 Fig — (TIF) [file pone.0154278.s005.tif]

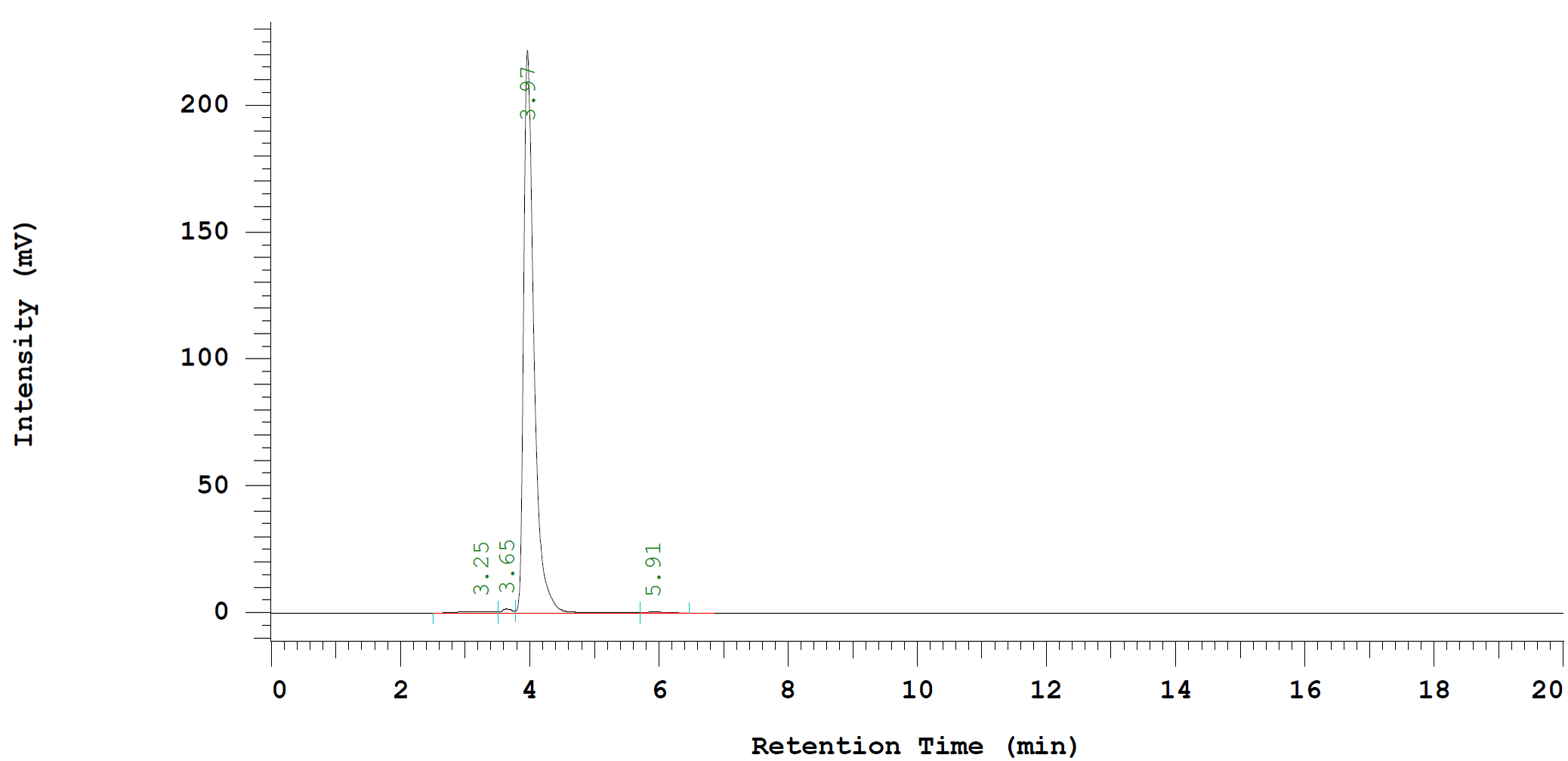

Supplement: S6 Fig — (TIF) [file pone.0154278.s006.tif]

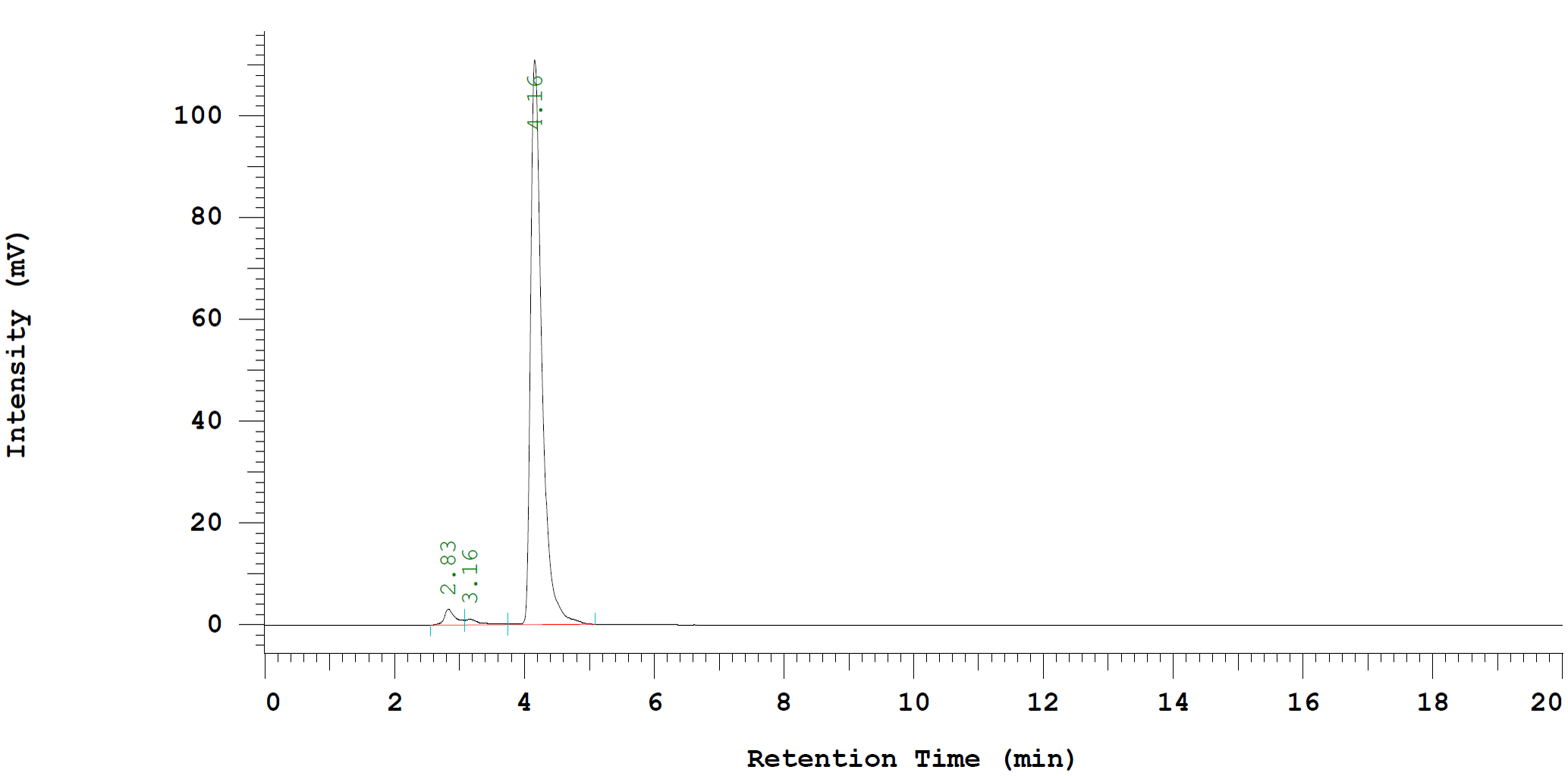

Supplement: S9 Fig — (TIF) [file pone.0154278.s009.tif]
