# Supplementary material for: Novel Anthra[1,2-c][1,2,5]Thiadiazole-6,11-Diones as Promising Anticancer Lead Compounds: Biological Evaluation, Characterization & Molecular Targets Determination
Source: PLoS One. 2016 Apr 21;11(4):e0154278. doi: 10.1371/journal.pone.0154278 (PMC4839570; doi:10.1371/journal.pone.0154278)
Supplement: S1 Table — a The average value of GI50 of every cell line panel tested in the five-dose NCI 60 cell line screen experiments. b The average value of GI50 of all of the tested cell lines in the five-dose NCI 60 cell line screen experiments and is equivalent to the mean graph midpoint (MID). c Compounds are rated as “selective” to the cell line panel if the ratio is more than 6, rated as “moderately selective” if the ratio is between 3 and 6, and rated as “non selective” if the ratio is less than 3. (DOCX) [file pone.0154278.s011.docx]

**Supporting Information**

**S1 Table.** Mean GI50 values (Molar) and selectivity ratios of NSC745885 and NSC757963 obtained from the NCI 60 cell line experiments.

| **The subpanel** | **NSC 745885** | | | **NSC 757963** | | |
| --- | --- | --- | --- | --- | --- | --- |
|  | **Mean GI_50_ of subpanel ^a^** | **Mean GI_50_ of all NCI-60 cell line panels ^b^** | **Selectivity ratio ^c^** | **Mean GI_50_ of subpanel ^a^** | **Mean GI_50_ of all NCI-60 cell line panels ^b^** | **Selectivity ratio ^c^** |
| **Leukemia** | 9.66E-07 | 3.23E-06 | 3.35 | 1.4E-06 | 1.09E-05 | 7.79 |
| **Non-Small Cell Lung Cancer** | 5.37E-06 |  | 0.6 | 2.07E-05 |  | 0.53 |
| **Colon Cancer** | 7.23E-06 |  | 0.45 | 1.14E-05 |  | 0.96 |
| **CNS Cancer** | 1.91E-06 |  | 1.69 | 7.71E-06 |  | 1.41 |
| **Melanoma** | 1.65E-06 |  | 1.96 | 8.44E-06 |  | 1.29 |
| **Ovarian Cancer** | 2.24E-06 |  | 1.44 | 6.77E-06 |  | 1.61 |
| **Renal Cancer** | 3.65E-06 |  | 0.89 | 1.85E-05 |  | 0.59 |
| **Prostate Cancer** | 2.45E-06 |  | 1.32 | 8.53E-06 |  | 1.28 |
| **Breast Cancer** | 1.54E-06 |  | 2.1 | 5.11E-06 |  | 2.14 |

^a^ The average value of GI_50_ of every cell line panel tested in the five-dose NCI-60 cell line screen experiments.

^b^ The average value of GI_50_ of all of the tested cell lines in the five-dose NCI-60 cell line screen experiments and is equivalent to the mean graph midpoint (MID).

^c^ Compounds are rated as “selective” to the cell line panel if the ratio is more than 6, rated as “moderately selective” if the ratio is between 3 and 6, and rated as “non selective” if the ratio is less than 3.
