# Supplementary material for: Novel Anthra[1,2-c][1,2,5]Thiadiazole-6,11-Diones as Promising Anticancer Lead Compounds: Biological Evaluation, Characterization & Molecular Targets Determination
Source: PLoS One. 2016 Apr 21;11(4):e0154278. doi: 10.1371/journal.pone.0154278 (PMC4839570; doi:10.1371/journal.pone.0154278)
Supplement: S2 Table — a The average value of GI50 of every cell line panel tested in the five-dose JFCR 39 cell line screen experiments. b The average value of GI50 of all of the tested cell lines in the five-dose JFCR 39 cell line screen experiments and is equivalent to the mean graph midpoint (MG-MID). c Compounds are rated as “selective” to the cell line panel if the ratio is more than 6, rated as “moderately selective” if the ratio is between 3 and 6, and rated as “non selective” if the ratio is less than 3. (DOCX) [file pone.0154278.s012.docx]

**Supporting Information**

**S2 Table.** Mean GI50 values (Molar) and selectivity ratios of NSC745885 and NSC757963 obtained from the JFCR 39 cell line experiments.

| **The subpanel** | **NSC 745885** | | | **NSC 757963** | | |
| --- | --- | --- | --- | --- | --- | --- |
|  | **Mean GI_50_ of subpanel ^a^** | **Mean GI_50_ of all JFCR-39 cell line panels ^b^** | **Selectivity ratio ^c^** | **Mean GI_50_ of subpanel ^a^** | **Mean GI_50_ of all JFCR-39 cell line panels ^b^** | **Selectivity ratio ^c^** |
| **Breast Cancer** | 1.66E-06 | 2.62E-06 | 1.58 | 1.72E-06 | 3.15385E-06 | 1.83 |
| **CNS Cancer** | 1.87E-06 |  | 1.4 | 2.00E-06 |  | 1.58 |
| **Colon** **Cancer** | 5.36E-06 |  | 0.49 | 5.94E-06 |  | 0.53 |
| **Lung Cancer** | 4.13E-06 |  | 0.63 | 5.86E-06 |  | 0.54 |
| **Melanoma** | 8.60E-07 |  | 3.04 | 1.9E-06 |  | 1.66 |
| **Ovarian Cancer** | 1.63E-06 |  | 1.6 | 2.08E-06 |  | 1.52 |
| **Renal Cancer** | 2.10E-06 |  | 1.25 | 1.75E-06 |  | 1.8 |
| **Stomach** **Cancer** | 1.71E-06 |  | 1.53 | 1.97E-06 |  | 1.6 |
| **Prostate Cancer** | 1.65E-06 |  | 1.59 | 2.05E-06 |  | 1.54 |

^a^ The average value of GI_50_ of every cell line panel tested in the five-dose JFCR-39 cell line screen experiments.

^b^ The average value of GI_50_ of all of the tested cell lines in the five-dose JFCR-39 cell line screen experiments and is equivalent to the mean graph midpoint (MG-MID).

^c^ Compounds are rated as “selective” to the cell line panel if the ratio is more than 6, rated as “moderately selective” if the ratio is between 3 and 6, and rated as “non selective” if the ratio is less than 3.
