# Supplementary material for: Novel Anthra[1,2-c][1,2,5]Thiadiazole-6,11-Diones as Promising Anticancer Lead Compounds: Biological Evaluation, Characterization & Molecular Targets Determination
Source: PLoS One. 2016 Apr 21;11(4):e0154278. doi: 10.1371/journal.pone.0154278 (PMC4839570; doi:10.1371/journal.pone.0154278)
Supplement: S3 Table — a Only compounds showing salient correlations with NSC745885 were selected and displayed in this table. Compounds appearing more than once in the COMPARE analysis results (due to difference in number of tested cell lines or in the hiConc of the compared experiments) were not included in this table. However the order of ranking of all of the compared compounds is retained. b This coefficient ranges from -1 to +1. Compounds with positive coefficient values approaching 1 have high similarities with the test compound, while those with negative coefficient values approaching -1 have high differences with the test compound. (DOCX) [file pone.0154278.s013.docx]

**Supporting Information**

**S3 Table.** NCI STANDARD AGENTS with similar activity profiles to NSC745885.^a^

| **GI_50_ analysis** | | | | **TGI analysis** | | | |
| --- | --- | --- | --- | --- | --- | --- | --- |
| **Compared compound** | **Pearson’s correlation coefficient ^b^** | **Mechanism of action category** | **Rank** | **Compared compound** | **Pearson’s correlation coefficient ^b^** | **Mechanism of action category** | **Rank** |
| Melphalan | 0.451 | Alkylating Agents | 1 | Dianhydrogalactitol | 0.547 | Alkylating Agents | 1 |
| Asaley | 0.425 | Alkylating Agents | 2 | Methyl CCNU (semustine) | 0.494 | Alkylating Agents | 2 |
| Fluorodopan | 0.394 | Alkylating Agents | 3 | AZQ (diaziquone) | 0.464 | Alkylating Agents | 3 |
| AZQ  (diaziquone) | 0.391 | Alkylating Agents | 4 | Piperazinedione | 0.449 | Alkylating Agents | 5 |
| CBDCA (carboplatin) | 0.382 | Alkylating Agents | 5 | Fluorodopan | 0.449 | Alkylating Agents | 6 |
|  | | | | Melphalan | 0.405 | Alkylating Agents | 8 |
|  | | | | Asaley | 0.39 | Alkylating Agents | 9 |
|  |  |  |  | N,N-dibenzyl daunomycin | -0.323 | Topoisomerase II Inhibitors | 1 |

^a^ Only compounds showing salient correlations with NSC745885 were selected and displayed in this table. Compounds appearing more than once in the COMPARE analysis results (due to difference in number of tested cell lines or in the hiConc of the compared experiments) were not included in this table. However the order of ranking of all of the compared compounds is retained.

b This coefficient ranges from -1 to +1. Compounds with positive coefficient values approaching 1 have high similarities with the test compound, while those with negative coefficient values approaching -1 have high differences with the test compound.
