# Supplementary material for: Novel Anthra[1,2-c][1,2,5]Thiadiazole-6,11-Diones as Promising Anticancer Lead Compounds: Biological Evaluation, Characterization & Molecular Targets Determination
Source: PLoS One. 2016 Apr 21;11(4):e0154278. doi: 10.1371/journal.pone.0154278 (PMC4839570; doi:10.1371/journal.pone.0154278)
Supplement: S5 Table — a This coefficient ranges from -1 to +1. Compounds with positive coefficient values approaching 1 have high similarities with the test compound, while those with negative coefficient values approaching -1 have high differences with the test compound. (DOCX) [file pone.0154278.s015.docx]

**Supporting Information**

**S5 Table.** JFCR drugs with similar activity profiles to NSC745885.

| **Compared compound** | **Pearson’s correlation coefficient ^a^** | **Molecular Targets / Drug Type** | **Rank** |
| --- | --- | --- | --- |
| Bleomycin | 0.417 | DNA strand break | 1 |
| E7070 (Indisulam) | 0.408 | Carbonic Anhydrase Type VB Inhibitors | 2 |
| Cisplatin | 0.39 | TNFSF6 Expression Inhibitors BIRC4 Expression Enhancers | 3 |

^a^ This coefficient ranges from -1 to +1. Compounds with positive coefficient values approaching 1 have high similarities with the test compound, while those with negative coefficient values approaching -1 have high differences with the test compound.
