# Supplementary material for: Novel Anthra[1,2-c][1,2,5]Thiadiazole-6,11-Diones as Promising Anticancer Lead Compounds: Biological Evaluation, Characterization & Molecular Targets Determination
Source: PLoS One. 2016 Apr 21;11(4):e0154278. doi: 10.1371/journal.pone.0154278 (PMC4839570; doi:10.1371/journal.pone.0154278)
Supplement: S7 Table — a This coefficient ranges from -1 to +1. Compounds with positive coefficient values approaching 1 have high similarities with the test compound, while those with negative coefficient values approaching -1 have high differences with the test compound, and a value of zero indicates no correlation at all. (DOCX) [file pone.0154278.s017.docx]

**Supporting Information**

**S7 Table.** JFCR drugs with similar activity profiles to NSC757963.

| **Compared compound** | **Pearson’s correlation coefficient ^a^** | **Molecular Targets / Drug Type** | **Rank** |
| --- | --- | --- | --- |
| 4-Hydroperoxycyclophosphamide | 0.307 | Alkylating drugs | 1 |
| E7070 (Indisulam) | 0.301 | CDK2 Inhibitors, Cyclin E Inhibitors Carbonic Anhydrase Type VB | 2 |
| 6-Mercaptopurine | 0.260 | Purine Antagonists | 3 |

^a^ This coefficient ranges from -1 to +1. Compounds with positive coefficient values approaching 1 have high similarities with the test compound, while those with negative coefficient values approaching -1 have high differences with the test compound, and a value of zero indicates no correlation at all.
